# Screening and tuberculosis preventive treatment among household contacts of drug-resistant tuberculosis patients: a scoping review

**DOI:** 10.1186/s12879-026-14056-1

**Published:** 2026-07-24

**Authors:** Siling Chen, Lan Luo, Fanghua Gong

**Affiliations:** 1 School of Nursing, Hunan Normal University, Changsha, China; 2 Kiang Wu Nursing College of Macau, Macau, China; 3 Department of Rehabilitation Medicine, Hunan Provincial People’s Hospital, The First Affiliated Hospital of Hunan Normal University, Changsha, China; 4 Hunan Tuberculosis Prevention and Control Institute (Hunan Chest Hospital), 519 Xianjiahu Road, Yuelu District, Changsha, 410013 China

**Keywords:** Drug-resistant tuberculosis, Household contacts, Screening, Tuberculosis preventive treatment, Scoping review

## Abstract

**Background:**

Drug-resistant tuberculosis (DR-TB) is associated with low treatment success rates and increased mortality. Household contacts (HHCs) face an elevated risk of infection and disease progression due to prolonged exposure. Consequently, systematic screening and tuberculosis preventive treatment (TPT) are critical to interrupt transmission within households. However, screening practices and TPT regimens vary substantially across countries and settings worldwide, and the available evidence remains fragmented. This study aims to synthesize evidence on screening strategies and TPT regimens for HHCs of patients with DR-TB, identify implementation barriers and research gaps, and inform future contact management approaches.

**Methods:**

We systematically searched nine databases including PubMed, Embase, and Web of Science. Each database was searched from inception to November 13, 2025. Eligible studies included observational studies (including cohort and cross-sectional studies), implementation studies, and randomized controlled trials reporting on screening approaches or TPT strategies among HHCs of patients with DR-TB. Data extraction was conducted in accordance with the Joanna Briggs Institute (JBI) methodological framework for scoping reviews, and reporting followed the Preferred Reporting Items for Systematic Reviews and Meta-Analyses for Scoping Reviews (PRISMA-ScR) guidelines. Two reviewers independently extracted data using a standardized form, including study characteristics, screening strategies, diagnostic methods, TPT regimens, safety and adherence outcomes, and implementation-related factors. The findings were summarized descriptively and presented as a structured narrative synthesis organized by thematic domains.

**Results:**

A total of 26 studies were included. Most studies (20/26) employed a sequential cascade screening strategy combining symptom assessment, imaging evaluation (when clinically indicated), and molecular or microbiological confirmation, often supplemented by tuberculin skin test (TST) or interferon-gamma release assays (IGRAs) for latent tuberculosis infection (LTBI) detection. Most studies were conducted in high TB burden low- and middle-income countries, particularly in Pakistan, South Africa, and China, with several multicenter studies spanning countries in Africa, Asia, and Latin America. Pediatric studies frequently incorporated gastric aspiration as an adjunct diagnostic method. TPT was implemented in 14 studies, with initiation rates ranging from 2% to 98.8%. Levofloxacin-based regimens were the predominant preventive treatment strategy. Compared with combination regimens, levofloxacin monotherapy generally achieved higher treatment completion rates (68.4%–100% vs. 56%–70.3%). Children experienced fewer adverse events than adults (10% vs. 37%). Frequently reported implementation barriers included resource limitations, transportation difficulties, stigma, and poor treatment adherence.

**Conclusion:**

Screening and TPT for HHCs of patients with DR-TB were commonly implemented using sequential cascade screening approaches and levofloxacin-based preventive treatment regimens across diverse settings. Included studies generally reported acceptable treatment completion and safety outcomes, particularly for levofloxacin-based monotherapy, although implementation outcomes varied substantially across regions and populations. Resource limitations, stigma, transportation difficulties, and poor adherence were frequently reported as barriers to implementation. Given the substantial heterogeneity in study design, screening strategies, and implementation contexts, findings should be interpreted cautiously. Further research is needed to improve evidence on standardized screening approaches, regimen effectiveness, and implementation feasibility across different settings, particularly among high-risk populations such as young children and people living with human immunodeficiency virus (HIV).

**Clinical trial number:**

Not applicable.

**Supplementary Information:**

The online version contains supplementary material available at 10.1186/s12879-026-14056-1.

## Introduction

Tuberculosis (TB) remains a significant global public health challenge, with the 2025 World Health Organization (WHO) Global TB Report documenting approximately 10.7 million incident cases and 1.23 million deaths in 2024 [1]. Despite standardized treatment regimens achieving cure rates of approximately 90%, suboptimal prescribing, poor adherence, and drug quality issues have driven the emergence of drug-resistant tuberculosis (DR-TB), including isoniazid-resistant tuberculosis, rifampicin-resistant (RR-TB), multidrug-resistant (MDR-TB), pre-extensively drug-resistant (pre-XDR-TB), and extensively drug-resistant (XDR-TB) strains [1]. Among an estimated 390,000 DR-TB patients globally in 2024, only approximately 40% received standardized treatment, with an overall treatment success rate below 75% [1]. This low treatment coverage leaves a substantial number of DR-TB patients undiagnosed or untreated, posing persistent risks of mortality and transmission [1]. High-burden countries, such as China, India, the Philippines, and Russia, account for more than half of the global multidrug-resistant and rifampicin-resistant tuberculosis (MDR/RR-TB) caseload, highlighting the concentrated geographic impact of this epidemic [1].

Household contacts (HHCs), defined as persons who shared the same enclosed living space with an index patient for one or more nights, or for frequent or extended daytime periods, during the three months before the start of the index patient’s current treatment [2], represent a high-risk population within the DR-TB transmission chain and remain insufficiently addressed in many high-burden settings. Compared with drug-susceptible tuberculosis (DS-TB), DR-TB is characterized by longer treatment durations, slower sputum culture conversion, and greater bacteriological positivity, collectively amplifying household transmission risk [3]. Molecular epidemiological studies have confirmed high strain concordance between index DR-TB cases and HHCs who subsequently developed disease, with resistance profiles frequently shared between index cases and contacts who developed active TB [4]. These findings support household transmission as a primary route of DR-TB dissemination. Epidemiological data indicate that approximately 37–39% of HHCs are diagnosed with latent tuberculosis infection (LTBI) [5]. Although LTBI is not infectious, it can reactivate during immune compromise to cause active disease and sustain transmission [6, 7].

Systematic screening and management of HHCs are therefore fundamental to interrupting secondary DR-TB transmission. While international guidelines have converged on this priority, they diverge substantially in recommended strategies, owing to heterogeneity in resource availability and epidemiological context. The 2024 WHO consolidated guidelines on tuberculosis preventive treatment (TPT) advocate systematic multi-modal screening (symptom assessment, immunological tests, imaging, and molecular assays) combined with a 6-month levofloxacin-based preventive treatment regimen [2]. The European Centre for Disease Prevention and Control (ECDC) surveillance data highlight substantial regional variation in the epidemiology and burden of DR-TB across European countries, underscoring the need for context-specific prevention and control strategies [8]. The 2025 joint guidelines from the American Thoracic Society (ATS), Centers for Disease Control and Prevention (CDC), European Respiratory Society (ERS), and Infectious Diseases Society of America (IDSA) explicitly classify DR-TB exposure as a systematic screening indication in children [9]. However, significant implementation gaps remain across settings with varying resource availability. High-income settings typically deploy interferon-gamma release assays (IGRAs) and molecular testing within structured contact management systems, whereas resource-constrained low- and middle-income countries (LMICs) rely predominantly on symptom screening and tuberculin skin tests (TSTs), resulting in suboptimal contact tracing completeness and TPT initiation rates [10–12].

TPT, defined as the administration of anti-tuberculosis drugs to individuals with LTBI or those at high risk, after excluding active disease, remains critically underutilized among HHCs of patients with DR-TB [13]. Globally, approximately 5.3 million high-risk individuals received TPT in 2024, yet coverage among HHCs reached only 25%, compared with 58% for people living with human immunodeficiency virus (HIV) [1]. Although isoniazid- and rifampicin-based regimens are effective for DS-TB contacts, they are unsuitable for contacts of DR-TB patients [2, 14]. The 2024 WHO recommendation of a 6-month levofloxacin-based preventive regimen for contacts of MDR/RR-TB patients has been subsequently validated by multiple randomized controlled trials (RCTs) and meta-analyses [2, 15–17]. Nonetheless, significant practical uncertainties persist, including increased adverse reaction risk with age, suboptimal tolerability and adherence among adults, and limited evidence on long-term safety, especially in children and people living with HIV across diverse resource settings [18].

These gaps highlight the need for a more systematic synthesis of existing evidence. Current studies on screening strategies, TPT regimens, and implementation barriers among HHCs of patients with DR-TB remain scattered across different settings and populations. As a result, it remains difficult to assess whether the available evidence is sufficient to inform policy and practice, to identify gaps between recommendations and real-world implementation, and to determine which populations and settings are insufficiently represented in current research and practice. These limitations provide a clear rationale for the present review.

A scoping review is an appropriate approach to address this gap. Compared with systematic reviews or meta-analyses, which are generally intended to answer focused questions based on relatively homogeneous evidence, scoping reviews are better suited to synthesizing diverse study designs, mapping existing evidence, and identifying research gaps in an evolving field [19–21]. Given the heterogeneity in study designs, populations, outcomes, and settings across the current literature, a scoping review can provide a comprehensive overview of existing evidence, identify key research gaps, and inform future evidence synthesis, clinical practice, and policy development.

This review primarily aims to map the implementation of screening and TPT programs for HHCs of patients with DR-TB across diverse settings. It seeks to characterize how these programs are delivered in practice, summarize reported outcomes, and identify the system level barriers and facilitators that influence implementation. The review is intended to provide evidence that may inform future clinical practice guidelines and public health policy. Specifically, this scoping review will: (1) describe the screening strategies and diagnostic tools employed; (2) describe the available TPT regimens, including their reported safety profiles and adherence outcomes; and (3) identify the main barriers and facilitating factors influencing implementation across different regions and populations. To this end, the review addresses the following research questions:

1. What screening strategies and detection methods have been used for HHCs of DR-TB patients in existing studies?
2. What TPT regimens are available for this population, and what are their reported implementation outcomes (effectiveness, safety, adherence)?
3. What are the primary barriers and facilitators influencing the implementation of screening and TPT programs?

## Methods

This scoping review was conducted in accordance with the methodological framework proposed by the Joanna Briggs Institute (JBI) [21, 22] and the Preferred Reporting Items for Systematic Reviews and Meta-Analyses extension for Scoping Reviews (PRISMA-ScR) guidelines [23]. The study protocol was prospectively registered in the Open Science Framework (OSF; Registration DOI: 10.17605/OSF.IO/CWMGN).

### Retrieval strategy

A comprehensive systematic search was performed across nine databases: PubMed, Embase, Web of Science, Cochrane Library, CINAHL, SinoMed, China National Knowledge Infrastructure (CNKI), Wanfang Database, and VIP Database. The search strategy combined Medical Subject Headings (MeSH), free-text terms and Boolean operators (AND/OR). The search strategy was adapted for each database according to its indexing system and search interface. Each database was searched from inception to November 13, 2025. The search terms and related variants used included “Household Contact”, “Drug-Resistant Tuberculosis”, “Family Close Contacts”, “Extensively Drug-Resistant Tuberculosis”, “Multidrug-Resistant Tuberculosis”, “Screening”, “Tuberculosis Preventive Treatment”. The full search strategies for PubMed and CNKI are provided in Supplementary Appendices S1 and S2, respectively. For Chinese-language databases (SinoMed, CNKI, Wanfang, VIP), searches were conducted using equivalent Chinese-language terms.

In addition to the systematic database searches, supplementary searches were conducted to identify potentially relevant gray literature. Google Scholar was searched using simplified keyword combinations (e.g., “drug-resistant tuberculosis” AND “household contacts” AND “preventive treatment”; “MDR-TB” AND “household contacts” AND “screening”), and the first 200 relevance-ranked records were screened because of platform limitations in accommodating complex search strategies. Chinese-language dissertations were retrieved through the thesis sub-database of CNKI, with document types restricted to doctoral and master’s dissertations, using equivalent Chinese-language search terms (e.g., drug-resistant tuberculosis, household contacts, preventive treatment). In addition, the reference lists of all included studies and relevant review articles identified during the search process were manually screened to identify any potentially eligible studies not captured through the electronic database searches.

### Eligibility criteria

#### Inclusion criteria

Since this is a scoping review, we used the PCC (Population / Concept / Context) framework recommended by JBI [22] to identify eligible studies as discussed below.

P (Population): HHCs of patients with DR-TB. Studies were eligible if the study population was identified as HHCs by the original study authors. Minor variations in the operational definition of HHCs across studies were permitted, consistent with the evidence-mapping purpose of this scoping review.

C (Concept): screening strategies, TPT measures, and adherence outcomes. Screening strategies, defined as any systematic approach to identify active TB disease or LTBI among HHCs, including clinical symptom assessment, imaging (chest radiography or computed tomography [CT]), microbiological testing (smear, culture, Xpert MTB/RIF), and immunological testing (TST or IGRA). TPT, operationalized as any pharmacological regimen administered to HHCs after active TB has been excluded, with the intent to prevent progression from LTBI to active disease [2]. Relevant TPT outcomes included treatment initiation rates, completion rates, safety profiles (adverse events and discontinuation), and effectiveness outcomes (incidence of active TB during follow-up). Adherence outcomes were defined according to the criteria reported in the original studies.

C (Context): residential household settings globally.

#### Exclusion criteria

1. Literature for which full text is unavailable.
2. Duplicate publications.
3. Literature not in Chinese or English.
4. Conference abstracts, which were only presented at domestic or international conferences but not publicly published.
5. Review articles, systematic reviews, conference papers, or other commentaries (editorials, commentaries).
6. Studies on DS-TB and general TB.

### Selection process

Two researchers with training in evidence-based methods independently conducted literature screening and data extraction. Prior to formal screening, both reviewers piloted the eligibility criteria on a random subset of 50 records retrieved from the database search to ensure consistent interpretation of the inclusion and exclusion criteria. Discrepancies were discussed and the screening criteria were further refined where necessary. The reviewers then independently screened titles and abstracts, followed by full-text assessment of potentially eligible studies. Any disagreements arising during the screening process were resolved through discussion and consensus, and unresolved cases were adjudicated by a third senior reviewer. The screening process was conducted as follows: all records retrieved from the databases were imported into EndNote X9. After duplicate records were removed, titles and abstracts were reviewed. A preliminary screening based on predefined inclusion and exclusion criteria was performed to identify potentially eligible studies. Full-text articles were subsequently reviewed for final eligibility. The two researchers cross-checked their results to determine the final included studies.

### Critical appraisal of included studies

To enhance interpretation of evidence derived from heterogeneous study designs, methodological quality was assessed using the JBI Critical Appraisal Tools according to study type [24, 25]. Separate appraisal checklists were applied for RCTs, cohort studies, case series, and cross-sectional studies. Each item was rated as “Yes,” “No,” “Unclear,” or “Not Applicable.” Two reviewers independently conducted the appraisal, and discrepancies were resolved through consultation with a third reviewer. Consistent with scoping review methodology, methodological quality was not used as a criterion for study exclusion, but was considered during interpretation of the findings. The appraisal findings were used to support interpretation of the evidence rather than to determine study eligibility. Study protocols were not subject to formal quality appraisal, as JBI critical appraisal tools are designed for completed studies reporting empirical results; where a protocol was identified as an associated publication of an included study, it was used solely to supplement methodological details not fully reported in the primary results paper.

### Data extraction

Two researchers independently extracted data using Excel spreadsheets in accordance with the study objectives. Data extraction covered author, publication year, country of origin (where the study was conducted), study type, study population, total number of index cases, number of HHCs, screening strategy, TPT regimen, implementation barriers, and facilitating factors (e.g., family support, health education, community-based follow-up, and programmatic support) related to screening or TPT implementation. The extracted data were descriptively summarized according to the study objectives, including screening strategies, diagnostic approaches, TPT regimens, implementation barriers, and facilitating factors. Key findings and study characteristics were synthesized narratively, and charts were developed to visualize the results. When multiple publications reported findings from the same cohort at different time points, these reports were included for completeness but were treated as a single cohort during evidence synthesis to avoid double counting.

## Results

### Search results

The database search yielded 1,868 records. After removal of duplicates, 1,232 records remained, of which 1,114 were excluded following title and abstract screening. Full-text review of the remaining 118 articles led to the exclusion of 92 studies (9 inappropriate study designs, 27 unavailable full texts, 47 irrelevant topics, and 9 reviews, commentaries, or case reports). Ultimately, 26 studies were included: 2 Chinese-language and 24 English-language publications. Supplementary searches through Google Scholar, CNKI dissertations, and manual reference screening did not identify any additional eligible studies.

**Fig. 1:**
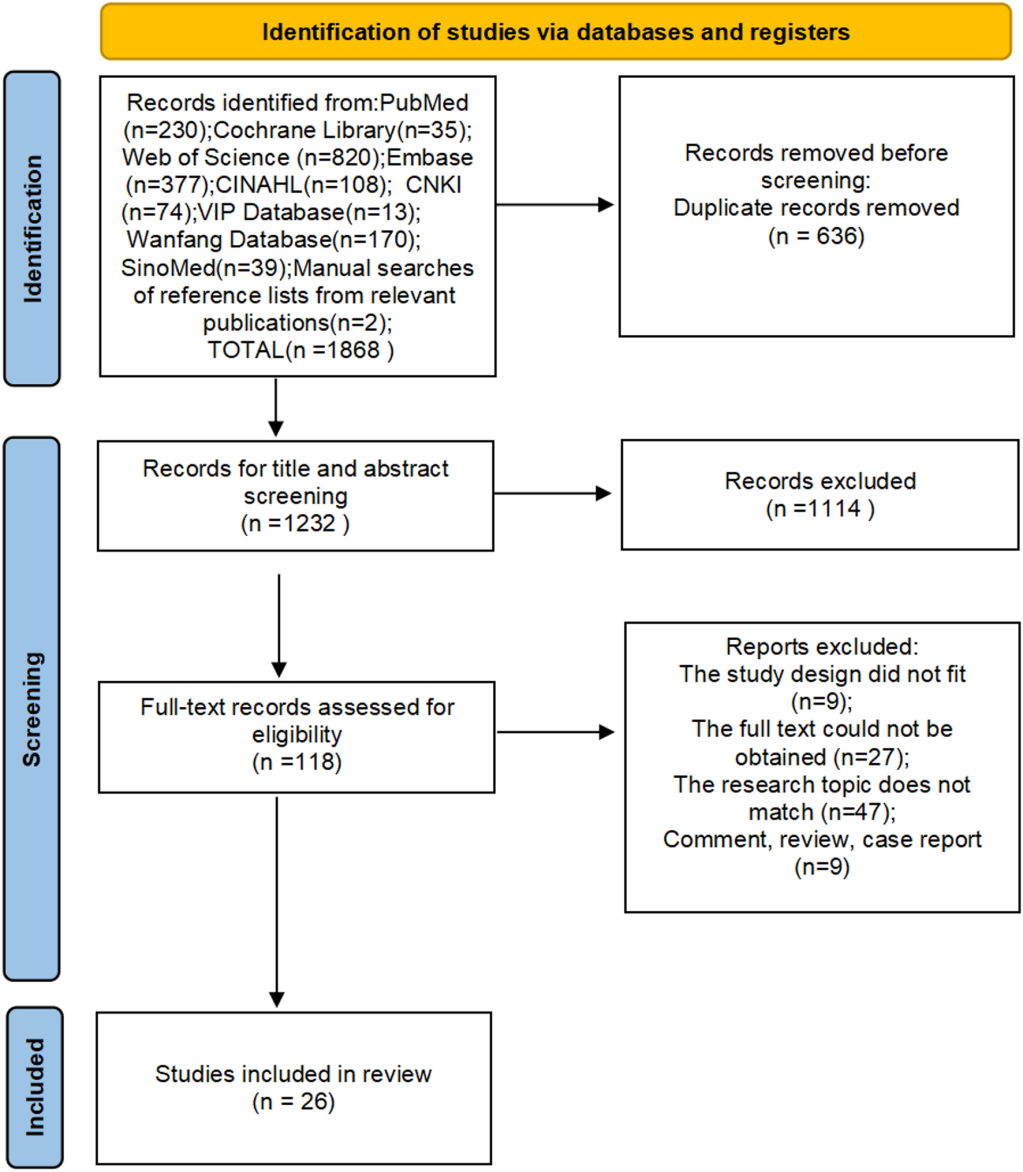
PRISMA flow-diagram for the scoping review

**Fig. 2:**
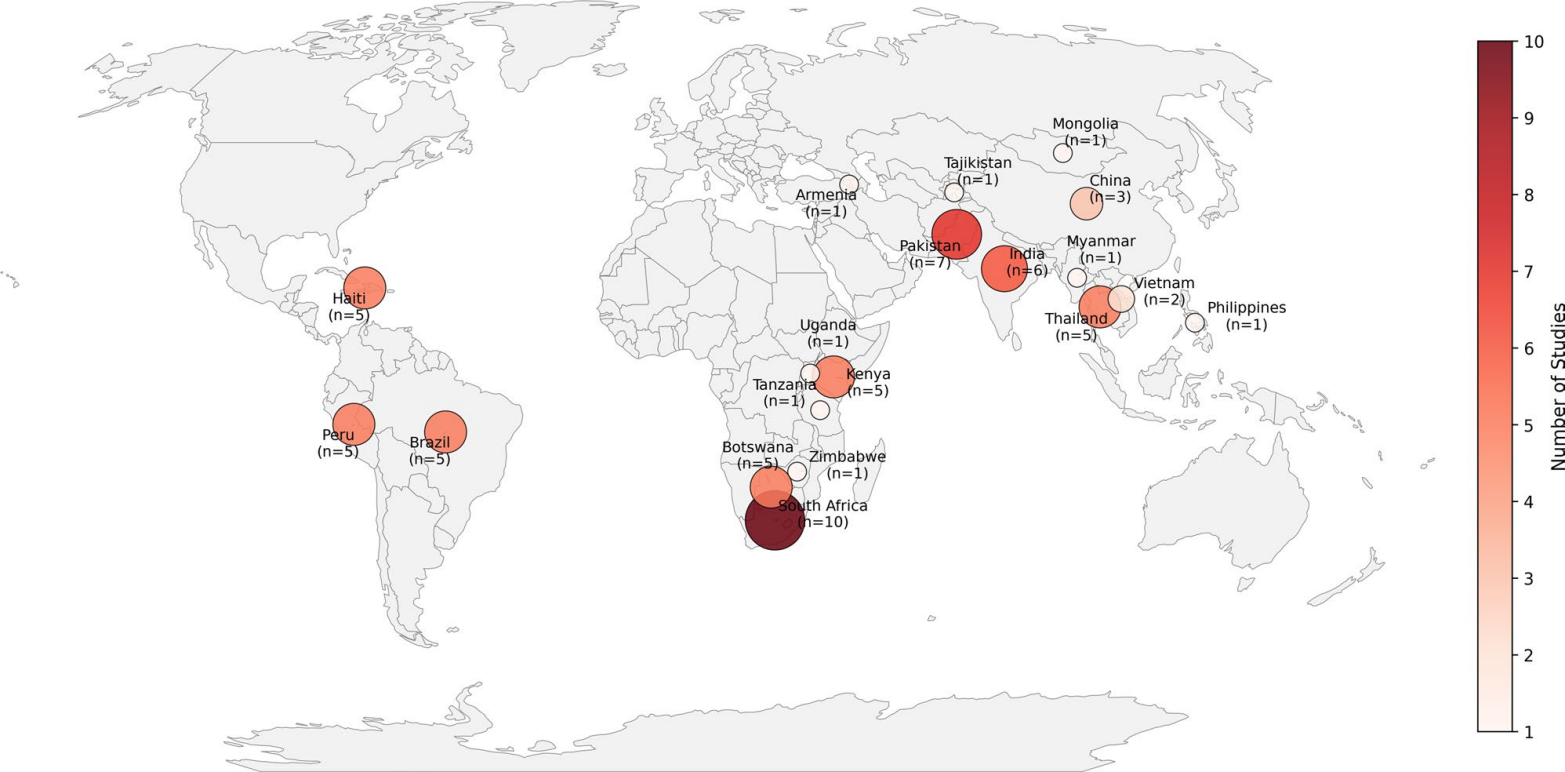
Global distribution of included studies

Geographically, three studies were conducted in China, seven in Pakistan, and five in South Africa. Four studies were multicenter investigations across eight high-burden countries (Botswana, Brazil, Haiti, India, Kenya, Peru, South Africa, and Thailand), and one multicenter study spanned 13 countries. The remaining six studies were conducted individually in Vietnam, Armenia, Tajikistan, India, Mongolia, and Myanmar. By design, the included studies comprised 8 prospective cohort studies, 1 prospective implementation study, 1 retrospective cohort study, 4 RCTs, 1 case series and 11 cross-sectional studies. The study selection process is shown in Fig. 1, study characteristics are summarized in Supplementary Table S1, and geographic distribution is shown in Fig. 2.

### Critical appraisal of included studies

Methodological quality was formally appraised for 24 of the 26 included studies, while two study protocols were excluded from appraisal as specified in the Methods [26, 27]. The results of the critical appraisal are presented in Supplementary Tables S2-S5. Overall, the included studies demonstrated moderate to high methodological quality. Both RCTs achieved “Yes” ratings across all 13 items of the JBI appraisal checklist, indicating a low risk of bias and strong methodological rigor. The cohort studies were generally methodologically robust; however, several studies demonstrated limitations in the identification and management of potential confounding factors, as well as incomplete follow-up reporting. The cross-sectional studies consistently applied appropriate inclusion criteria and outcome measurement methods, although inadequate adjustment for confounding was identified in several studies. These methodological limitations and potential sources of bias were taken into consideration during the interpretation and synthesis of the review findings.

###  HHC screening strategies

#### Screening algorithms

Across the 26 included studies, the predominant approach was a sequential cascade screening strategy, employed in approximately 20 studies. This multi-step approach began with symptom assessment (evaluating cough, fever, night sweats, and weight loss), followed by chest radiography in non-pregnant contacts with clinical indications, and subsequently by microbiological confirmatory testing, including sputum smear microscopy, mycobacterial culture, and molecular assays such as Xpert MTB/RIF, for those with radiographic abnormalities or persistent symptoms. This cascade was often supplemented by immunological testing (TST or IGRA) to enable concurrent detection of active TB and LTBI. Chest radiography was the most frequently used screening tool, appearing in 24 of 26 studies (92.3%). The frequency of all individual screening methods is summarized in Table 1.

Additional diagnostic and laboratory approaches were reported in several studies. HIV testing was incorporated in nine studies [26, 28–33], genotyping was conducted in one study from China [34]. Laboratory inflammatory markers, including erythrocyte sedimentation rate, were used in three studies [29, 35, 36], and chest CT was applied in two studies [36, 37].

**Fig. 3:**
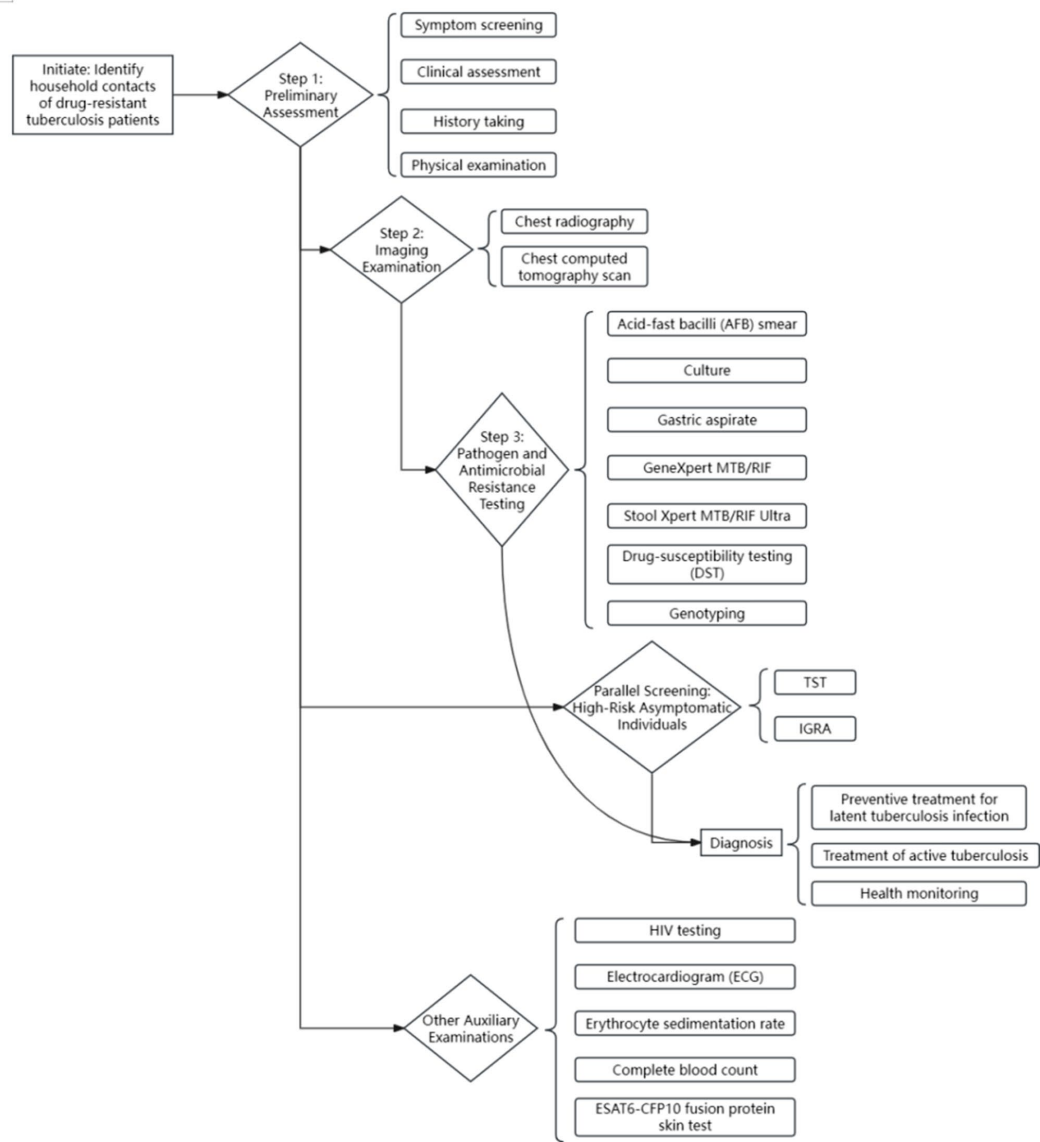
Flowchart of screening strategy classification

In pediatric cohorts, gastric aspiration was used as an adjunct microbiological sampling method in six studies [26, 28–30, 36, 38], with additional follow-up data reported from the Schaaf cohort [35]. Stool Xpert MTB/RIF Ultra testing was utilized in two studies [30, 32]. Drug-susceptibility testing (DST) of index case isolates was performed in five studies to guide individualized regimen selection [34, 36, 38–40]. A classification of screening strategy types is presented in Fig. 3.

**Table 1:** Frequency of screening methods used across included studies

Screening method	Number of studies (*N* = 26)	Frequency (%)
Chest radiography	24	92.3
Symptom screening	21	80.8
AFB smear	18	69.2
GeneXpert MTB/RIF	17	65.4
TST	16	61.5
Culture	13	50.0
IGRA	11	42.3
HIV testing	9	34.6
Gastric aspirate	6	23.1
Physical examination	6	23.1
DST	5	19.2
History taking	3	11.5
Chest computed tomography scan	2	7.7
Stool Xpert MTB/RIF Ultra	2	7.7
Erythrocyte sedimentation rate	2	7.7
Complete blood count	2	7.7
Clinical assessment	2	7.7
ECG	1	3.8
Genotyping	1	3.8
ESAT6-CFP10 fusion protein skin test	1	3.8

Abbreviations: AFB, acid-fast bacilli; TST, tuberculin skin test; IGRA, interferon-gamma release assay; DST, drug-susceptibility testing; ECG, electrocardiogram; ESAT6-CFP10, early secreted antigenic target 6-culture filtrate protein 10

#### Screening initiation timing and follow-up frequency

The timing of screening initiation varied considerably across studies. Ten study cohorts did not report specific initiation times [29, 31, 32, 36, 37, 39, 41–44]. Among those that did, screening was initiated immediately or within one week in four studies conducted in China, Mongolia, Myanmar, and South Africa [33, 34, 45, 46]; within two weeks in Tajikistan [47]; at two to four weeks in Pakistan [40]; and at eight to nine weeks in the large multicenter studies across eight high-burden countries and in the Vietnamese RCT [28, 38, 48–50]. Two South African pediatric studies initiated screening within six months of index case diagnosis [27, 30], and one study protocol specified completion within 30 days of index case enrolment, extendable to 70 days for suspected active TB [26].

All included studies conducted baseline screening at enrollment, followed by periodic reassessments during follow-up. Follow-up frequency varied widely: from monthly clinic visits [36] to annual evaluations [34]. Several studies implemented high-frequency monitoring, such as monthly home visits combined with clinic assessments every two months during treatment [31]. Other studies used prespecified multi-point schedules (e.g., at 2, 8, 24, 48, and 96 weeks [26, 27, 30, 45, 48]) or simplified protocols of assessments every two to three months [32, 35], every six months [39, 46, 47], or as a single concentrated review [29].

#### LTBI diagnostic approaches

Immunological testing for LTBI was incorporated in 20 of the 26 studies. Nine unique study cohorts employed TST alone [29, 31, 34, 36, 40, 43, 45, 48, 51]; four used IGRA alone [27, 33, 38, 49]; and seven applied both methods in combination [26, 28, 32, 37, 39, 47, 50]. The choice of testing modality was closely linked to regional resource availability. Studies conducted in resource-limited settings such as Pakistan, China, Armenia, and Tajikistan predominantly used TST. In contrast, the large multicenter studies and the South African pediatric RCTs used IGRA alone or in combination with TST.

TST positivity thresholds varied across studies, introducing a source of heterogeneity in LTBI prevalence estimates. In adult populations, cut-off values of ≥ 5 mm [34, 39] and ≥ 10 mm [43, 48] were most commonly applied. In pediatric cohorts, a threshold of ≥ 15 mm was used in the South African Schaaf cohort studies [29] and subsequent follow-up report [35] to enhance specificity in a Bacillus Calmette-Guérin (BCG)-vaccinated population. Some studies adjusted thresholds based on BCG vaccination history, although the proportion of participants undergoing BCG scar examination was not consistently reported. IGRA results were interpreted per manufacturer instructions in all applicable studies. Diagnostic criteria for active TB were consistently based on microbiological confirmation (positive culture or molecular testing) combined with clinical or radiographic features; in pediatric cases, a clinical diagnosis based on chest X-ray findings indicative of hilar lymphadenitis was also applied [45].

LTBI prevalence estimates varied substantially across regions and populations, ranging from 27% in a South African pediatric cohort [33] to 72% in a multicenter feasibility study across eight high-burden countries [28] and reaching 99.8% in a study from Vietnam [48]. This heterogeneity reflects differences in population characteristics, local transmission intensity, and diagnostic methods applied.

#### Active TB detection and screening outcomes across regions

Active TB cumulative incidence among HHCs varied widely across studies, ranging from 0.7% to 35.8%. Studies employing systematic cascade screening in adult or mixed-age populations generally reported incidence rates of 1.1%–12% [28, 38]. In contrast, studies using symptom-driven, patient-initiated approaches, in which only symptomatic contacts sought care, detected substantially higher rates: up to 35.8% among symptomatic contacts in India, with MDR-TB accounting for 22.9% of active TB cases identified [43].

Regional variation was pronounced. Cross-sectional studies from Pakistan reported relatively high detection rates of 5.95%–21.6% [41, 44], while studies from China reported rates of 1.4%–9.8% [34, 37, 39], and large multicenter studies spanning eight high-burden countries reported intermediate rates of 2.3%–12% [28, 50]. The study from Tajikistan, which included the largest sample (6,654 HHCs), reported a relatively low cumulative incidence of 0.7%, likely reflecting the structured national contact tracing program in that setting [47].

Children under five years of age consistently showed higher TB burden than older contacts. Multiple cohort studies reported baseline TB disease prevalence of approximately 10%–12% in this age group [29, 30, 46]. Long-term follow-up of the South African Schaaf cohort showed that cumulative TB incidence reached 23% over 30 months among young child contacts [35], while Amanullah et al. [36] in Pakistan reported a cumulative TB rate of 7.5% among children aged 0–14 years. Several studies reported relatively high proportions of individuals refusing further testing or children not being brought for follow-up assessments by their caregivers [36, 37].

Screening coverage was reported in 14 of 26 studies. Seven studies reported coverage of ≥ 90% [31, 35, 40, 43, 45, 46, 50], three reported ≥ 80% [38, 39, 51], and four reported ≥ 70% [28, 30, 32, 44]. The remaining 12 studies did not explicitly report coverage rates.

###  TPT

#### TPT provision rates

Of the 26 included publications, 14 studies [26–31, 33, 35, 38, 40, 43, 45, 48, 51] described TPT implementation, representing 13 independent cohorts. The remaining 12 studies either did not implement TPT or did not report relevant data; this gap was concentrated among Chinese studies [34, 37, 39] and Pakistani cross-sectional surveys [41, 42, 44].

TPT initiation rates varied markedly by setting and study design. Cross-sectional studies across high-burden countries reported coverage among adult contacts of only 2%–4% [28, 38, 50], with rates of 20% among children under five and only 13% among HIV-positive contacts in one multicenter feasibility study [38], despite both groups being considered high-priority populations. These rates contrast sharply with prospective cohort and intervention studies, which reported initiation rates of 80%–98.8% in Pakistan [31, 40, 51], Vietnam [48], and South Africa [30, 33, 35]. Under a community health worker-supported model in Mongolia, 43% of TST-positive children received TPT [45].

#### TPT regimens

##### Monotherapy

Levofloxacin was the most commonly used monotherapy, employed in five of the 14 studies reporting TPT [27, 30, 33, 45, 48]. The standard regimen consisted of once-daily administration for six months, with an adult dose of 10–15 mg/kg and a pediatric dose of 15–20 mg/kg (maximum 750 mg/day) [27, 33, 48]. This regimen was implemented across diverse settings, including a large community-based double-blind RCT in Vietnam (*n* = 3,948) [48], two pediatric cluster-randomized trials in South Africa [27, 33], a mixed-age cohort study in South Africa [30], and a prospective implementation study in Mongolia [45].

Isoniazid monotherapy was reported in two studies. In Apolisi et al. [30], 9.5% of contacts (those with RR-TB exposure only) received daily isoniazid for six months. Shadrach et al. [43] prescribed isoniazid monotherapy to LTBI-positive contacts in an Indian cohort. Neither study reported completion rates or safety outcomes for this subgroup separately.

Delamanid monotherapy was reported in two studies. The PHOENIx trial protocol specified weight-based dosing over 26 weeks (2.5–12 kg: 30 mg; 12–20 kg: 50 mg; 20–25 kg: 100 mg; 25–30 kg: 150 mg; ≥30 kg: 200 mg), but outcome data are not yet available [26]. In the South African cohort of Apolisi et al. [30], 7.4% of contacts with fluoroquinolone-resistant TB exposure received delamanid for six months at an unspecified dose; completion was not reported separately.

##### Combination therapy

Combination therapy was described in seven unique study cohorts [26, 28, 29, 31, 38, 40, 51], primarily as fluoroquinolone-based dual regimens or individualized multidrug regimens guided by index case DST results.

Fluoroquinolone-based dual regimens (predominantly levofloxacin or moxifloxacin combined with ethambutol or ethionamide) were the main strategy in three prospective Pakistani cohorts [31, 40, 51], where 80% of eligible contacts initiated a six-month dual regimen. This approach reduced the risk of progression to active TB by 65% within two years (incidence rate ratio [IRR] = 0.35, 95% CI: 0.14–0.87) [31], with a cumulative active TB incidence of 1.2% among treated contacts [51]. The ethionamide-containing variant carried a 2.1-fold higher risk of adverse events than the ethambutol-containing regimen, particularly gastrointestinal symptoms and dizziness [51].

Individualized multidrug regimens (2–4 drugs) guided by index case resistance profiles were reported in four studies [26, 28, 29, 38]. In the South African Schaaf cohort, children receiving appropriate DST-guided chemoprophylaxis had substantially lower TB incidence than untreated children (5% vs. 20%; OR 4.97, 95% CI 1.06–23.33) [35], demonstrating a significant protective effect. In contrast, 90.5% of contacts in a multinational study discontinued combination therapy within one year [38], and coverage in routine-care settings was below 1% in another feasibility study [28], illustrating the significant gap between protocol efficacy and real-world uptake.

#### Adherence and treatment completion

Treatment completion rates differed systematically between monotherapy and combination therapy. Levofloxacin monotherapy studies reported completion rates of 68.4%–100% [27, 30, 33, 45, 48]. In the Mongolian community health worker model, all children who initiated treatment completed the full course (100%) [45]; 80% of children completed treatment in the South African cohort [30]; and 85.3% of children in the TB-CHAMP trial received ≥ 90% of the prescribed dose [33]. In the Vietnamese RCT, 68.4% of 2,041 contacts completed ≥ 80% of doses [48]. In contrast, fluoroquinolone-based combination regimens achieved completion rates of 56%–70.3% across Pakistani and South African cohorts [31, 35, 40, 51].

Treatment discontinuation due to adverse events was generally low. In the Vietnamese RCT, 7.4% permanently discontinued levofloxacin due to adverse reactions, and 23.2% discontinued for any reason (vs. 9.1% in the placebo group) [48]. In the South African pediatric trial, only 0.7% of children under five discontinued due to adverse events [33]. For combination regimens, 6.4% discontinued due to adverse reactions in the Pakistani cohort [51], and 24% of South African children were lost to follow-up before completing individualized multidrug regimens [35].

The mode of treatment delivery consistently influenced completion rates. In Mongolia, community health worker-supported home delivery achieved 100% completion in children [45]. In Pakistan, a directly observed therapy (DOT) model combining home visits with volunteer supervision achieved a 12-month completion rate of 96% [40, 51]. These findings suggest that the delivery model may be as important as the regimen in determining overall completion.

### Special populations

#### Pediatric contacts

Twenty-two publications reported pediatric data, of which three independent cohorts specifically targeted children aged ≤ 5 years [27, 29, 33], with additional 30-month follow-up data from the Schaaf cohort [35]. Drug dosing was weight-stratified across all pediatric studies: levofloxacin was prescribed at 15–20 mg/kg/day in children under five years of age and at 7.5–10 mg/kg/day in older children and adolescents [27, 33, 40, 48]; delamanid used a five-tier weight-band strategy [26].

Children under five demonstrated a consistently more favorable safety profile compared with adults. Adverse event incidence was 10% in this age group versus 37% in adults over 19 years [51]. Gastrointestinal symptoms were the most frequently reported adverse effects. Grade 3–4 serious adverse events were rare, with a treatment discontinuation rate due to adverse events of only 0.7% [33]. No acquired fluoroquinolone resistance was detected among children in any study reporting this outcome [30, 35, 51]. Monotherapy completion rates in children (80%–100%) consistently exceeded those for combination regimens (56%–70.3%) [30, 31, 33, 35, 40, 45].

#### HIV-positive contacts

Data on HIV-positive contacts were limited across the included studies. HIV-positive contacts were enrolled in twelve studies [26, 28, 30, 33, 35, 38, 43, 46–50]; however, HIV-stratified TPT outcomes were rarely reported. Available evidence suggests that TPT regimens and dosing in HIV-positive contacts were largely consistent with those used in the general contact population, and no study reported the use of differentiated or specialized management protocols for this subgroup [28, 30, 33, 38, 46, 50].

### Implementation factors

#### Barriers

Implementation barriers were consistently identified across geographic settings but varied in their relative prominence. At the patient and family level, refusal of screening or TPT was reported in studies from China, Pakistan, and multinational settings [28, 31, 32, 39, 42, 43]: one Chinese study found that 17.9% of contacts declined immunological testing [39], and pregnant or breastfeeding women voluntarily discontinued medication [39]. Poor treatment adherence was attributed to adverse drug reactions, prolonged treatment duration, daily pill burden, and absence of perceived illness in asymptomatic contacts [40, 48]. In pediatric populations, adherence was further impaired by large tablet size, unpleasant taste, and the need for forced administration [27, 33]. Caregivers of young children were more likely to refuse blood sampling and were less likely to bring children for follow-up visits [32, 44, 50]. Sociocultural factors, including reliance on traditional medicine and stigma-associated avoidance, were more prominent in studies from Pakistan and sub-Saharan Africa [28, 33, 49].

At the health service provider level, insufficient knowledge and limited confidence in initiating TPT for DR-TB contacts were reported in studies from Mongolia and China [37, 45]. Limited diagnostic capacity for childhood TB, together with the absence of child-specific protocols and national preventive treatment guidelines, was identified as a major barrier in Pakistan, Myanmar, and multi-country settings [42, 44, 50]. Gastric aspirate or induced sputum for microbiological evaluation was only used in a small number of studies [26, 28–30, 36, 38], and DST results from index cases were not consistently applied to guide pediatric regimen selection [29]. Drug and reagent supply instability also constrained program implementation; ethambutol shortages in Pakistan necessitated substitution with less-tolerable ethionamide [40, 51].

At the health system level, resource constraints, including workforce shortages, equipment limitations, and high costs of systematic radiographic screening, were widely reported [30, 35, 46, 50]. Symptom-based initial screening demonstrated limited sensitivity (approximately 38%), increasing the risk of missed LTBI and early active TB [40]. Contact tracing was largely confined to household members, with limited coverage of broader high-risk networks [43, 44]. In some routine-care settings, TPT coverage was as low as 0.1% [49].

At the social and environmental level, TB-related stigma was consistently documented in studies from South Africa, Pakistan, Mongolia, and multi-country settings [28, 45–47, 49, 50]. Socioeconomic disadvantages such as poverty, overcrowding, and substandard housing further increased transmission risk and reduced treatment adherence. Households affected by MDR-TB typically had larger family sizes and higher poverty levels than the general population [43, 44, 47, 49].

#### Facilitators

Decentralized and community-based service delivery models consistently improved screening coverage and treatment completion. In Mongolia, community health workers (CHWs)-led home visits achieved initial screening coverage of 99.4% and 12-month retention exceeding 98% [45]. In Pakistan, a DOT model combining home visits with volunteer supervision achieved a 12-month completion rate of 96% [40, 51]. In South Africa, increased home visit frequency improved participation among healthy children in clinic-based programs [30]. Facility-based verbal screening was reported as feasible and high-yield in Pakistan [42], and transportation subsidies with free medication provision reduced financial barriers in multiple settings [40, 44, 45].

Integration of contact management into national tuberculosis programs (NTPs) provided institutional support for sustained implementation. In Myanmar, MDR-TB care was incorporated into national planning with dedicated township nurses and volunteer networks [46]. Pakistan’s NTP oversaw the full MDR-TB management pathway [44]. Multicenter collaborative networks and centralized contact databases facilitated expanded coverage and program sustainability [26, 39, 50]. Electronic drug monitoring tools and online coordination platforms improved adherence management in settings with digital infrastructure [26, 45]. High-quality clinical research platforms, such as the PHOENIx trial, contributed to standardization and quality assurance in multicenter implementation [26].

The implementation barriers and facilitating factors are summarized in Supplementary Table S6.

## Discussion

HHCs of DR-TB patients are chronically exposed to airborne *Mycobacterium tuberculosis* and represent a high-risk group for LTBI and secondary transmission [5]. Establishing standardized screening procedures and implementing effective TPT may therefore help interrupt DR-TB transmission and support progress toward achieving the WHO goal of “Ending Tuberculosis” [52, 53]. Evidence from the included studies suggests that HHC screening coverage is generally high in well-resourced and intervention-study settings, with common approaches beginning with symptom assessment and incorporating comprehensive strategies that include chest radiography, sputum smear microscopy, Xpert MTB/RIF molecular testing, and either the TST or IGRA [45, 54]. Substantial regional variation exists in TPT availability. Levofloxacin remains the most commonly used monotherapy, whereas fluoroquinolone-based dual-drug regimens are the predominant combination therapy [15, 48]. TPT dosing in children is typically weight-based, with safety and tolerability profiles that appeared more favorable in younger children than in adults across the studies that reported comparative data [27, 33, 48, 51]. Regimens for HIV-infected contacts are largely consistent with those for the general population [2, 33, 38, 40].

Substantial heterogeneity was observed across study designs, geographic settings, and implementation contexts. Most evidence on screening strategies was derived from observational studies, while the limited RCTs focused primarily on TPT efficacy rather than comparative evaluation of screening algorithms [12]. In addition, studies were concentrated in a small number of high-burden countries, with limited representation from several regions with substantial DR-TB burden, potentially restricting the broader applicability of findings [1]. Considerable variation also existed in diagnostic thresholds, follow-up duration, and definitions of key outcomes, contributing to inconsistencies in reported estimates of LTBI prevalence, TPT uptake, and active TB incidence across studies [5]. These methodological and contextual differences limited direct comparability between studies and highlight the need for more standardized approaches in future DR-TB contact research.

The choice of screening tool is closely related to regional TB burden and the availability of healthcare resources. High-burden or resource-limited settings tend to favor TST, whereas high-income settings or contexts requiring minimal BCG interference more frequently adopt IGRA [2, 55, 56]. The preference for TST may be due to its low cost and ease of administration; however, it requires a follow-up visit for interpretation after 48–72 h and is susceptible to interference from BCG vaccination and exposure to non-tuberculous mycobacteria [2, 57]. IGRA, by contrast, is unaffected by BCG vaccination, provides objective interpretation, and requires only a single blood draw. However, its use necessitates higher laboratory standards, careful sample transport, and greater testing costs [2, 56]. Therefore, in resource-limited settings, TST remains a practical screening tool, whereas IGRA offers advantages in settings with adequate resources where diagnostic specificity is prioritized, consistent with evidence demonstrating its superior overall diagnostic accuracy [57].

A clear distinction exists between the evidence supporting screening strategies and that supporting TPT effectiveness in HHCs of patients with DR-TB. Evidence on screening approaches, including screening algorithms, diagnostic methods, and timing of evaluation, was derived from observational studies and therefore remains primarily descriptive and context-dependent [58]. In contrast, evidence for TPT effectiveness is supported by recent randomized trials, including TB-CHAMP and VQUIN, which demonstrated reduced active TB incidence following levofloxacin-based preventive therapy in both children and adults [15, 17, 48]. This difference in evidence strength should be considered when interpreting findings related to screening practices and TPT regimens.

Several included studies reported relatively high rates of TB among young child HHCs, particularly those under five years of age [29, 36]. In South Africa, Schaaf et al. [29] observed a baseline TB disease prevalence of 12% among children aged ≤ 5 years, which reached 23% during the 30-month follow-up period [35]. Similarly, Amanullah et al. [36] reported a TB rate of 7.5% among children aged 0–14 years in Pakistan. These findings are in line with previous evidence suggesting that young children are at heightened risk of TB following MDR-TB exposure [59, 60]. Taken together, these observations highlight the importance of early screening and close follow-up of young child HHCs, particularly in high-burden settings.

Regarding infection screening, no unified standard currently exists for the positive threshold of the TST in children, leading to detection rates ranging from 23.8% to 58.7% across studies. Schaaf et al. [29] reported a detection rate of 51.6% using a threshold of ≥ 15 mm, whereas Huerga et al. [32] applied a lower threshold of ≥ 10 mm and observed a detection rate of 47.1% in children under five years of age. These discrepancies suggest that the diagnostic performance of TST is influenced by threshold selection, BCG vaccination history, and exposure to environmental non-tuberculous mycobacteria [61]. In high-prevalence or high-BCG-coverage settings, raising the cutoff value can reduce false positives but may increase the risk of missed diagnoses, whereas in lower-risk settings a higher cutoff improves specificity and reduces unnecessary TPT. A two-step approach, using TST followed by IGRA, may enhance overall diagnostic accuracy while maintaining sensitivity [61]. Nevertheless, national guidelines differ regarding threshold settings and target populations [61], indicating that standardization of TST interpretation criteria for pediatric populations across different epidemiological contexts remains an unresolved challenge.

Secondary cases predominantly develop symptoms within one year of exposure to index cases [34, 35, 38, 39]. These patterns suggest that early screening initiation and sustained follow-up over at least two years may be important for timely case detection. The timing of screening among HHCs of individuals with DR-TB varies substantially across studies, primarily influenced by factors such as index case diagnosis timing, laboratory result delays, imaging and microbiological testing requirements, healthcare system capacity, and national policy regulations [45, 47, 54, 62–65]. International guidelines, including the WHO consolidated guidelines on TPT [2] and the WHO consolidated guidelines on systematic screening for TB disease [54], along with national TB guidance documents [66], recommend initiating HHC screening promptly after laboratory confirmation of DR-TB in the index case to ensure timely evaluation of exposed contacts. Some countries, including Tajikistan and Mongolia, explicitly require completion within 14 days [45, 47]. In practice, however, the initiation time ranges from immediate screening to several months after diagnosis. Delayed initiation may result in missed concurrent cases, for example, Yang et al. [39] reported five HHCs detected one month after the index case confirmation, highlighting the risk of pre-diagnosis household transmission. Overall, prompt screening initiation is associated with earlier detection of LTBI and active TB, which in turn may support timely TPT initiation and potentially reduce the risk of disease progression and household transmission, although direct causal evidence from experimental studies on screening timing is lacking [52, 54, 62–64].

The TPT provision rate exhibits substantial regional variation, consistent with the findings of Giridharan et al. [67]. These differences are primarily driven by policy, resource allocation, and sociocultural factors. Countries with high HIV prevalence often implement TPT earlier due to the elevated risk of HIV-TB co-infection, and variations in national guidelines regarding contact eligibility and preferred treatment regimens directly affect TPT provision rates [67, 68]. In resource-limited settings, low TPT initiation rates are often attributable to insufficient drug supplies, workforce shortages, and transportation barriers, whereas in relatively well-resourced regions, free medication provision and community health worker support can improve both initiation and completion rates [69]. Sociocultural factors and family acceptance, including stigma, concerns about medication, and caregiver willingness, further influence TPT uptake and adherence [28, 49].

For HHCs of DR-TB, current evidence suggests that fluoroquinolone monotherapy, particularly levofloxacin, may offer advantages over combination regimens in adherence, safety, feasibility, and treatment coverage. Completion rates for combination therapy ranged from 56% to 70.3% [31, 35, 40, 51], whereas levofloxacin monotherapy achieved completion rates of 68.4%–100%. The rate of permanent treatment discontinuation due to adverse events was below 8%, and the incidence of serious adverse events was less than 3% [27, 30, 33, 35, 45, 48, 51]. In addition, monotherapy regimens are simpler to administer and may facilitate integration into routine contact management systems in high-burden and resource-constrained settings [30]. The 2024 WHO consolidated guidelines on TPT recommend daily levofloxacin for six consecutive months for contacts of MDR/RR-TB patients [2]. Consistently, joint guidelines from the ATS, CDC, ERS, and IDSA recommend prioritizing fluoroquinolone monotherapy based on the antimicrobial susceptibility profile of the index case isolate, with combination regimens considered only under specific resistance patterns [9]. These guidelines further state that, due to the high incidence of adverse reactions associated with pyrazinamide, it should not be used as a routine second drug in combination regimens [70]. For pediatric patients, levofloxacin is preferred because a solution formulation is available [70].

Although combination regimens demonstrated protective effects in certain high-exposure or historical cohorts, their implementation in high-burden settings is limited by low treatment completion, frequent adverse events, and substantial heterogeneity in regimen composition. Krishnan et al. [38] reported that differences in drug availability across countries resulted in fragmented, context-specific multidrug regimens with limited comparability of outcomes, and most contacts discontinued treatment within one year. Ethionamide-containing regimens were associated with poorer tolerability [35, 51], while individualized multidrug approaches were further constrained by dependence on DST results and local drug availability [31, 35, 40, 51]. In pediatric populations, weight-based dosing and increased medication burden may further reduce adherence to prolonged preventive treatment [40]. In China, no standardized TPT regimen is currently available for close contacts of MDR-TB patients; individualized preventive treatment may be implemented by selecting drugs to which the index case isolate is susceptible, based on drug resistance profiles [66].

Potential antimicrobial resistance risks associated with fluoroquinolone-based TPT remain an important concern in DR-TB contact management. Given the central role of fluoroquinolones in MDR/RR-TB treatment and the existing global burden of fluoroquinolone-resistant TB [1, 18], widespread preventive use could theoretically contribute to resistance selection. However, no acquired fluoroquinolone resistance was reported among pediatric contacts receiving levofloxacin-based TPT in studies that monitored this outcome [30, 35, 51]. Current evidence on resistance surveillance remains limited, particularly in adults and in settings with a high prevalence of fluoroquinolone resistance. In addition, levofloxacin-based TPT is unlikely to be appropriate for contacts exposed to fluoroquinolone-resistant MDR-TB, highlighting the need for further evaluation of alternative preventive regimens such as delamanid [26].

Evidence on TPT outcomes among HIV-positive HHCs of patients with DR-TB remains limited. Although several studies included HIV-positive contacts, outcomes were rarely reported separately according to HIV status, and none evaluated differentiated management strategies for this high-risk subgroup [28, 30, 33, 38, 46, 50]. This evidence gap is important given the increased risk of TB progression among people living with HIV and their prioritization for TPT in current guidelines [2]. Among the limited studies reporting TPT initiation according to HIV status, coverage among HIV-positive contacts remained low [38]. Levofloxacin-based TPT may offer practical advantages in HIV co-infected populations because it avoids the drug-drug interactions associated with rifamycin-based regimens and antiretroviral therapy [18]. However, evidence regarding its safety and effectiveness in HIV-positive DR-TB contacts remains insufficient, highlighting the need for future studies to report outcomes separately according to HIV status.

Economic feasibility remains an underexplored aspect of systematic screening and TPT implementation for HHCs of patients with DR-TB in resource-limited settings. None of the included studies reported formal cost-effectiveness analyses, although external modeling studies suggest that levofloxacin-based TPT may be cost-effective and potentially cost-saving in high-burden settings [18, 71, 72]. However, successful implementation depends not only on regimen efficacy, but also on health system capacity, including drug supply, diagnostic infrastructure, contact tracing, and adherence support.

A substantial gap was observed between TPT outcomes achieved under intensive research conditions and those reported in routine programmatic settings, highlighting an important implementation challenge in DR-TB contact management. Studies conducted in well-resourced research settings reported markedly higher TPT initiation and completion rates than routine-care programs [31, 38, 40, 49, 51], suggesting that implementation effectiveness is strongly influenced by health system capacity and service delivery models. Community-based and decentralized approaches, particularly those involving CHWs, appeared to improve screening coverage and treatment completion in several high-burden settings [40, 45]. These findings indicate that scalability depends not only on effective preventive regimens, but also on the integration of contact management into existing health systems, including adequate workforce support, supply-chain stability, and follow-up capacity. Future implementation research should place greater emphasis on evaluating practical delivery strategies and implementation outcomes in routine-care settings.

### Strengths

The primary strengths of this review include a systematic search across multiple databases, strict adherence to established methodological frameworks and the PRISMA-ScR guidelines [22, 23], and the inclusion of studies with diverse designs from multiple geographic regions. This study provides a comprehensive evidence map for HHCs screening and preventive treatment among DR-TB patients. The findings help identify current evidence gaps and provide directions for future research and policy development.

### Limitations

Several limitations of this review should be acknowledged. First, the included studies did not provide sufficient cost-effectiveness data, limiting evaluation of the economic feasibility and implementation value of DR-TB contact management strategies across different settings. Second, because this review primarily included published literature, some evidence from surveillance systems, registries, or programmatic reports may not have been captured. Third, substantial clinical and methodological heterogeneity across studies, including differences in screening algorithms, TPT regimens, follow-up duration, and outcome definitions, limited direct comparability between studies and precluded quantitative synthesis [19]. Finally, definitions of HHCs and exposure assessment methods were not fully consistent across studies, which may have contributed to variability in reported outcomes.

### Recommendations for future practice and research

Based on the findings of this review, several recommendations for future practice and research can be proposed.

First, future studies should optimize diagnostic cascades and implementation pathways for HHC management. Approximately 30–40% of HHCs discontinue further immunological testing after symptom screening, substantially reducing the effectiveness of screening programs [36, 37]. Future interventions should therefore be developed within scientifically grounded implementation frameworks to strengthen the continuity of the “screening–diagnosis–assessment–intervention” pathway. In children, TB diagnosis remains heavily dependent on invasive procedures, contributing to poor adherence and delayed detection [29, 36]. Large prospective cohort studies are urgently needed to validate the diagnostic performance of non-invasive tools, including stool Xpert Ultra and urine lipoarabinomannan (LAM), particularly among children under 5 years of age [73, 74]. In addition, standardized interpretation algorithms for TST and IGRA are needed to address inconsistencies in diagnostic thresholds and improve the balance between sensitivity and specificity [61].

Second, stronger clinical evidence is needed to guide preventive treatment strategies for HHCs of patients with DR-TB. No randomized trial has directly compared levofloxacin monotherapy with combination regimens, and evidence remains limited for HIV-positive contacts and contacts exposed to fluoroquinolone-resistant MDR-TB [17, 18]. Future studies should evaluate pharmacokinetics, safety, and drug interactions in vulnerable populations, including pregnant women, people living with HIV, and young children [26]. In addition, incorporating whole-genome sequencing (WGS) into longitudinal surveillance may help distinguish household transmission from community-acquired infection and better characterize acquired drug resistance [3, 4].

Third, health economic evaluation and international collaboration should be strengthened. Although emerging modeling evidence suggests that levofloxacin-based TPT may be cost-saving in high-burden settings [71, 72], locally contextualized cost-effectiveness analyses are needed across diverse resource settings to guide program investment decisions. Establishing international multicenter data-sharing platforms with standardized reporting protocols could accelerate evidence generation and policy translation, and would be particularly valuable for documenting long-term outcomes and AMR surveillance data [26].

## Conclusion

This scoping review maps the available evidence on screening strategies and TPT for HHCs of patients with DR-TB across diverse settings. Sequential cascade screening combined with TST or IGRA was the most commonly reported approach for identifying active TB and LTBI, while levofloxacin-based monotherapy was the predominant TPT regimen and generally showed favorable tolerability and treatment completion, particularly in children. However, substantial heterogeneity in population characteristics, screening algorithms, diagnostic criteria, and implementation capacity limited direct comparability across study settings, while resource limitations, stigma, and poor adherence were commonly reported as barriers to implementation. Early contact screening and timely TPT initiation may benefit high-risk groups, especially young children and people living with HIV. Further comparative studies are needed to improve evidence on standardized screening approaches, regimen effectiveness, and implementation strategies for DR-TB contact management.

## Supplementary Information

Below is the link to the electronic supplementary material.

Supplementary Material 1

## Data Availability

All data generated or analysed during this study are included in this published article.

## Abbreviations

TB: Tuberculosis
WHO: World Health Organization
DR-TB: Drug-resistant tuberculosis
RR-TB: Rifampicin-resistant tuberculosis
MDR-TB: Multidrug-resistant tuberculosis
pre-XDR-TB: Pre-extensively drug-resistant
XDR-TB: Extensively drug-resistant tuberculosis
DS-TB: Drug-susceptible tuberculosis
HHC: Household contact
LTBI: Latent tuberculosis infection
ATS: American Thoracic Society
ECDC: European Centre for Disease Prevention and Control
CDC: Centers for Disease Control and Prevention
ERS: European Respiratory Society
IDSA: Infectious Diseases Society of America
IGRA: Interferon-gamma release assay
LMIC: Low- and middle-income country
TST: Tuberculin skin test
TPT: Tuberculosis preventive treatment
HIV: Human immunodeficiency virus
RCT: Randomized controlled trial
JBI: Joanna Briggs Institute
PRISMA-ScR: Preferred Reporting Items for Systematic Reviews and Meta-Analyses for Scoping Reviews
CNKI: China National Knowledge Infrastructure
MeSH: Medical Subject Headings
TBI: Tuberculosis infection
TBD: Tuberculosis disease
DST: Drug-susceptibility testing
AFB: Acid-fast bacilli
BCG: Bacillus Calmette-Guérin
ECG: Electrocardiogram
IRR: Incidence rate ratio
CHW: Community health worker
DOT: Directly observed therapy
NTP: National Tuberculosis Program
LAM: Lipoarabinomannan
WGS: Whole-genome sequencing

## References

[1] World Health Organization. Global tuberculosis report 2025. 2025. https://www.who.int/teams/global-programme-on-tuberculosis-and-lung-health/tb-reports/global-tuberculosis-report-2025. Accessed 4 Dec 2025.

[2] World Health Organization. WHO consolidated guidelines on tuberculosis Module 1: prevention-tuberculosis preventive treatment, second edition. 2024. https://www.who.int/publications/i/item/9789240096196. Accessed 4 Dec 2025. 39298638

[3] Liu D, Huang F, Li Y, Mao L, He W, Wu S, et al. Transmission characteristics in Tuberculosis by WGS: nationwide cross-sectional surveillance in China. Emerg Microbes Infect. 2024;13(1):2348505. 38686553 10.1080/22221751.2024.2348505 PMC11097701

[4] Chiang SS, Brooks MB, Jenkins HE, Rubenstein D, Seddon JA, Water BJ, et al. Concordance of Drug-resistance Profiles Between Persons With Drug-resistant Tuberculosis and Their Household Contacts: A Systematic Review and Meta-analysis. Clin Infect Dis. 2021;73(2):250–63. 32448887 10.1093/cid/ciaa613 PMC8427728

[5] Akalu TY, Clements ACA, Gebreyohannes EA, Gilmour B, Alene KA. Prevalence of tuberculosis infection among contacts of drug-resistant tuberculosis patients: A systematic review and meta-analysis. J Infect. 2024;89(2):106198. 38906264 10.1016/j.jinf.2024.106198

[6] Kiazyk S, Ball TB. Latent tuberculosis infection: An overview. Can Commun Dis Rep. 2017;43(3–4):62–6. 29770066 10.14745/ccdr.v43i34a01 PMC5764738

[7] Zhang Y, Li H, Ma Z, Wang J, Yin X. Advances in imaging studies of active pulmonary tuberculosis. Radiol Infect Dis. 2023;10(4):125–9.

[8] European Centre for Disease Prevention and Control, WHO Regional Office for Europe. Tuberculosis surveillance and monitoring in Europe 2022–2024 data. 2024. https://www.ecdc.europa.eu/sites/default/files/documents/TB_Surveillance_Report_2024.pdf. Accessed 4 Dec 2025.

[9] Saukkonen JJ, Duarte R, Munsiff SS, Winston CA, Mammen MJ, Abubakar I, et al. Updates on the treatment of drug-susceptible and drug-resistant tuberculosis: an official ATS/CDC/ERS/IDSA clinical practice guideline. Am J Respir Crit Care Med. 2025;211(1):e1–34. 40693952 10.1164/rccm.202410-2096ST PMC11755361

[10] Bennani K, Van Den Boom M, ElMedrek MG, Hutin Y. Progress in programmatic management of drug-resistant TB, WHO Eastern Mediterranean Region, 2018–2023. IJTLD Open. 2024;1(9):404–10. 39301137 10.5588/ijtldopen.24.0348 PMC11409170

[11] Mpimbaza MM, Migisha R, Twesigomwe G, Bajunirwe F. Missed opportunity for tuberculosis screening and prevention and the associated factors among child contacts in rural southwestern Uganda. BMC Pulm Med. 2025;25(1):86. 41029609 10.1186/s12890-025-03906-4 PMC12487121

[12] Fox GJ, Barry SE, Britton WJ, Marks GB. Contact investigation for tuberculosis: a systematic review and meta-analysis. Eur Respir J. 2013;41(1):140–56. 22936710 10.1183/09031936.00070812 PMC3533588

[13] Tayal A, Kabra SK. Tuberculosis Preventive Treatment. Indian J Pediatr. 2024;91(8):823–9. 38095783 10.1007/s12098-023-04969-z

[14] World Health Organization. Latent tuberculosis infection: updated and consolidated guidelines for programmatic management. 2018. https://apps.who.int/iris/handle/10665/260233. Accessed 4 Dec 2025. 30277688

[15] Hesseling AC, Purchase SE, Martinson NA, Fairlie L, Schaaf HS, Brigden J, et al. Levofloxacin Preventive Treatment in Children Exposed to MDR Tuberculosis. N Engl J Med. 2024;391(24):2315–26. 39693542 10.1056/NEJMoa2314318

[16] Zhou G, Luo S, He J, Chen N, Zhang Y, Cai S, et al. Effectiveness and safety of tuberculosis preventive treatment for contacts of patients with multidrug-resistant tuberculosis: a systematic review and meta-analysis. Clin Microbiol Infect. 2024;30(2):189–96. 37741621 10.1016/j.cmi.2023.09.015

[17] Duong T, Brigden J, Simon Schaaf H, Garden F, Marais BJ, Anh Nguyen T, et al. A Meta-Analysis of Levofloxacin for Contacts of Multidrug-Resistant Tuberculosis. NEJM Evid. 2025;4(1):EVIDoa2400190. 39693627 10.1056/EVIDoa2400190

[18] Matteelli A, Lovatti S, Rossi B, Rossi L. Update on multidrug-resistant tuberculosis preventive therapy toward the global tuberculosis elimination. Int J Infect Dis. 2025;155:107849. 39993523 10.1016/j.ijid.2025.107849

[19] Munn Z, Peters MDJ, Stern C, Tufanaru C, McArthur A, Aromataris E. Systematic review or scoping review? Guidance for authors when choosing between a systematic or scoping review approach. BMC Med Res Methodol. 2018;18(1):143. 30453902 10.1186/s12874-018-0611-x PMC6245623

[20] Arksey H, O’malley L. Scoping studies: towards a methodological framework. Int J Soc Res Methodol. 2005;8(1):19–32.

[21] Peters MDJ, Marnie C, Tricco AC, Pollock D, Munn Z, Alexander L, et al. Updated methodological guidance for the conduct of scoping reviews. JBI Evid Synth. 2020;18(10):2119–26. 33038124 10.11124/JBIES-20-00167

[22] Joanna Briggs Institute. Scoping reviews. 2024. https://jbi-global-wiki.refined.site/space/MANUAL/355862497/10.+Scoping+reviews. Accessed 6 Dec 2025.

[23] Tricco AC, Lillie E, Zarin W, O’Brien KK, Colquhoun H, Levac D, et al. PRISMA Extension for Scoping Reviews (PRISMA-ScR): Checklist and Explanation. Ann Intern Med. 2018;169(7):467–73. 30178033 10.7326/M18-0850

[24] Joanna Briggs Institute. Critical Appraisal Tools. https://jbi.global/critical-appraisal-tools. Accessed 20 May 2026.

[25] Barker TH, Stone JC, Sears K, Klugar M, Leonardi-Bee J, Tufanaru C, et al. Revising the JBI quantitative critical appraisal tools to improve their applicability: an overview of methods and the development process. JBI Evid Synth. 2023;21(3):478–93. 36121230 10.11124/JBIES-22-00125

[26] Kendall MA, Hughes MD, Kim S, Aaron LA, Naini L, Suryavanshi N, et al. Protecting households on exposure to newly diagnosed index multidrug-resistant tuberculosis patients: Study protocol for the PHOENIx phase 3 clinical trial. Contemp Clin Trials. 2025;158:108084. 40992540 10.1016/j.cct.2025.108084 PMC12709595

[27] Seddon JA, Garcia-Prats AJ, Purchase SE, Osman M, Demers AM, Hoddinott G, et al. Levofloxacin versus placebo for the prevention of tuberculosis disease in child contacts of multidrug-resistant tuberculosis: study protocol for a phase III cluster randomised controlled trial (TB-CHAMP). Trials. 2018;19(1):693. 30572905 10.1186/s13063-018-3070-0 PMC6302301

[28] Gupta A, Swindells S, Kim S, Hughes MD, Naini L, Wu X, et al. Feasibility of Identifying Household Contacts of Rifampin-and Multidrug-resistant Tuberculosis Cases at High Risk of Progression to Tuberculosis Disease. Clin Infect Dis. 2020;70(3):425–35. 30942853 10.1093/cid/ciz235 PMC7188224

[29] Schaaf HS, Vermeulen HA, Gie RP, Beyers N, Donald PR. Evaluation of young children in household contact with adult multidrug-resistant pulmonary tuberculosis cases. Pediatr Infect Dis J. 1999;18(6):494–500. 10391177 10.1097/00006454-199906000-00004

[30] Apolisi I, Cox H, Tyeku N, Daniels J, Mathee S, Cariem R, et al. Tuberculosis Diagnosis and Preventive Monotherapy Among Children and Adolescents Exposed to Rifampicin-Resistant Tuberculosis in the Household. Open Forum Infect Dis. 2023;10(3):ofad087. 36910692 10.1093/ofid/ofad087 PMC10003730

[31] Malik AA, Gandhi NR, Lash TL, Cranmer LM, Omer SB, Ahmed JF, et al. Effectiveness of Preventive Therapy for Persons Exposed at Home to Drug-Resistant Tuberculosis, Karachi, Pakistan. Emerg Infect Dis. 2021;27(3):805–12. 33624580 10.3201/eid2703.203916 PMC7920671

[32] Huerga H, Sanchez-Padilla E, Melikyan N, Atshemyan H, Hayrapetyan A, Ulumyan A, et al. High prevalence of infection and low incidence of disease in child contacts of patients with drug-resistant tuberculosis: a prospective cohort study. Arch Dis Child. 2019;104(7):622–8. 30523172 10.1136/archdischild-2018-315411 PMC6589461

[33] Duong T, Brigden J, Purchase SE, Martinson NA, Fairlie L, Staples S, et al. Factors Predictive of Early Discontinuation of Preventive Treatment in Children With Household Exposure to Multidrug-resistant Tuberculosis. Open Forum Infect Dis. 2025;12(8):ofaf425. 40874184 10.1093/ofid/ofaf425 PMC12378377

[34] Zhou Y, Zhou M, Peng J, Duan Q, Chen J, Zhou M, et al. Analysis of household transmission of multidrug-resistant pulmonary tuberculosis. Chin J Antituberc. 2023;45(2):195–9.

[35] Schaaf HS, Gie RP, Kennedy M, Beyers N, Hesseling PB, Donald PR. Evaluation of young children in contact with adult multidrug-resistant pulmonary tuberculosis: a 30-month follow-up. Pediatrics. 2002;109(5):765–71. 11986434 10.1542/peds.109.5.765

[36] Amanullah F, Ashfaq M, Khowaja S, Parekh A, Salahuddin N, Lotia-Farrukh I, et al. High tuberculosis prevalence in children exposed at home to drug-resistant tuberculosis. Int J Tuberc Lung Dis. 2014;18(5):520–7. 24903786 10.5588/ijtld.13.0593

[37] Shi Z, Peng J, Li X, Fu X, Zou L, Chen Q, et al. Mycobacterium tuberculosis infection status and associated factors among household close contacts of rifampicin-resistant pulmonary tuberculosis patients: A single-center cross-sectional study. J Clin Tuberc Other Mycobact Dis. 2025;41:100561. 41019099 10.1016/j.jctube.2025.100561 PMC12476092

[38] Krishnan S, Wu X, Kim S, McIntire K, Naini L, Hughes MD, et al. 1-Year Incidence of Tuberculosis Infection and Disease Among Household Contacts of Rifampin- and Multidrug-Resistant Tuberculosis. Clin Infect Dis. 2023;77(6):892–900. 37227925 10.1093/cid/ciad301 PMC10681643

[39] Yang H, Ma J, Ren F, Li R, Wu Y, Zhang Y. Clinical characteristics of household contacts developing active pulmonary tuberculosis among 15 patients with drug-resistant pulmonary tuberculosis. Chin J Antituberc. 2023;45(2):172–80.

[40] Malik AA, Fuad J, Siddiqui S, Amanullah F, Jaswal M, Barry Z, et al. Tuberculosis Preventive Therapy for Individuals Exposed to Drug-resistant Tuberculosis: Feasibility and Safety of a Community-based Delivery of Fluoroquinolone-containing Preventive Regimen. Clin Infect Dis. 2020;70(9):1958–65. 31190072 10.1093/cid/ciz502

[41] Iqbal Z, Rameen Y, Anjum T, Hidayat U, Younis F. Infection and Disease among household contacts of Multidrug Resistant Tuberculosis Patients in Khyber Pakhtunkhwa. Pakistan J Chest Med. 2023;29(4):495–500.

[42] Qadeer E, Fatima R, Haq MU, Yaqoob A, Kyaw NTT, Shah S, et al. Yield of facility-based verbal screening amongst household contacts of patients with multi-drug resistant tuberculosis in Pakistan. J Clin Tuberc Other Mycobact Dis. 2017;7:22–7. 31723697 10.1016/j.jctube.2017.01.004 PMC6850254

[43] Shadrach BJ, Kumar S, Deokar K, Singh GV, Hariharan, Goel R. A study of multidrug resistant tuberculosis among symptomatic household contacts of MDR-TB patients. Indian J Tuberc. 2021;68(1):25–31. 33641847 10.1016/j.ijtb.2020.09.030

[44] Javaid A, Khan MA, Khan MA, Mehreen S, Basit A, Khan RA, et al. Screening outcomes of household contacts of multidrug-resistant tuberculosis patients in Peshawar, Pakistan. Asian Pac J Trop Med. 2016;9(9):909–12. 27633308 10.1016/j.apjtm.2016.07.017

[45] Tsogt B, Sambuu T, Purevsuren Y, Munkhbaatar S, Batsuren P, Khandaa OE, et al. Multidrug-resistant tuberculosis household contact screening and management by community healthcare workers in Mongolia: a prospective implementation study. Lancet Reg Health West Pac. 2025;63:101704. 41127709 10.1016/j.lanwpc.2025.101704 PMC12537571

[46] Kyaw NTT, Sithu A, Satyanarayana S, Kumar AMV, Thein S, Thi AM, et al. Outcomes of Community-Based Systematic Screening of Household Contacts of Patients with Multidrug-Resistant Tuberculosis in Myanmar. Trop Med Infect Dis. 2019;5(1):2. 31881646 10.3390/tropicalmed5010002 PMC7157714

[47] Rekart ML, Aung A, Cullip T, Mulanda W, Mun L, Pirmahmadzoda B, et al. Household drug-resistant TB contact tracing in Tajikistan. Int J Tuberc Lung Dis. 2023;27(10):748–53. 37749832 10.5588/ijtld.23.0066 PMC10519379

[48] Fox GJ, Nhung NV, Cam Binh N, Hoa NB, Garden FL, Benedetti A, et al. Levofloxacin for the Prevention of Multidrug-Resistant Tuberculosis in Vietnam. N Engl J Med. 2024;391(24):2304–14. 39693541 10.1056/NEJMoa2314325

[49] Kim S, Hesseling AC, Wu X, Hughes MD, Shah NS, Gaikwad S, et al. Factors associated with prevalent Mycobacterium tuberculosis infection and disease among adolescents and adults exposed to rifampin-resistant tuberculosis in the household. PLoS ONE. 2023;18(3):e0283290. 36930628 10.1371/journal.pone.0283290 PMC10022776

[50] Swindells S, Gupta A, Kim S, Hughes MD, Sanchez J, Mave V, et al. Resource utilization for multidrug-resistant tuberculosis household contact investigations (A5300/I2003). Int J Tuberc Lung Dis. 2018;22(9):1016–22. 30092866 10.5588/ijtld.18.0163 PMC6104641

[51] Malik AA, Becerra MC, Lash TL, Cranmer LM, Omer SB, Fuad J, et al. Risk Factors for Adverse Events in Household Contacts Prescribed Preventive Treatment for Drug-resistant Tuberculosis Exposure. Clin Infect Dis. 2021;72(10):1709–15. 32266942 10.1093/cid/ciaa327 PMC8315482

[52] World Health Organization. Global tuberculosis report 2024. 2024. https://www.who.int/publications/i/item/9789240101531. Accessed 20 May 2026.

[53] World Health Organization. WHO consolidated guidelines on tuberculosis: module 1: prevention: tuberculosis preventive treatment. 2020. https://www.who.int/publications/i/item/who-consolidated-guidelines-on-tuberculosis-module-1-prevention-tuberculosis-preventive-treatment. Accessed 20 May 2026.

[54] World Health Organization. WHO consolidated guidelines on tuberculosis: module 2: screening: systematic screening for tuberculosis disease. 2024. https://www.who.int/publications/i/item/9789240022676. Accessed 16 Dec 2025. 33822560

[55] Sharma SK, Vashishtha R, Chauhan LS, Sreenivas V, Seth D. Comparison of TST and IGRA in Diagnosis of Latent Tuberculosis Infection in a High TB-Burden Setting. PLoS ONE. 2017;12(1):e0169539. 28060926 10.1371/journal.pone.0169539 PMC5218498

[56] Centers for Disease Control and Prevention (CDC). Clinical Testing Guidance for Tuberculosis: Interferon Gamma Release Assay. 2024. https://www.cdc.gov/tb/hcp/testing-diagnosis/interferon-gamma-release-assay.html. Accessed 16 Dec 2025.

[57] Tobaiqi MA, Alshamrani MN, Sriram S, Mahmoud AB, Fadlalmola HA, Albadrani M. Interferon-Gamma Release Assays Versus Tuberculin Skin Test for Active Tuberculosis Diagnosis: A Systematic Review and Diagnostic Meta-Analysis. Diagnostics (Basel). 2025;15(18):2343. 41008717 10.3390/diagnostics15182343 PMC12468246

[58] Shah NS, Yuen CM, Heo M, Tolman AW, Becerra MC. Yield of contact investigations in households of patients with drug-resistant tuberculosis: systematic review and meta-analysis. Clin Infect Dis. 2014;58(3):381–91. 24065336 10.1093/cid/cit643 PMC3890332

[59] Martinez L, Cords O, Horsburgh CR, Andrews JR, Pediatric TB, Contact Studies Consortium. The risk of tuberculosis in children after close exposure: a systematic review and individual-participant meta-analysis. Lancet. 2020;395(10228):973–84. 32199484 10.1016/S0140-6736(20)30166-5 PMC7289654

[60] Purchase SE, Brigden J, Seddon JA, Martinson NA, Fairlie L, Staples S, et al. Risk of Tuberculosis Infection in Young Children Exposed to Multidrug-resistant Tuberculosis in the TB-CHAMP Multi-site Randomized Controlled Trial. Clin Infect Dis. 2025;81(5):e401–9. 40440402 10.1093/cid/ciaf284 PMC12728287

[61] Jia J, Chen D, Liu L, Siddiqui MJ, Yang F, Zhu Y, et al. Prevalence of Latent Tuberculosis Infection Among Healthy Young Children and Adolescents and a Two-step Approach for the Diagnosis of Tuberculosis Infection in Chengdu, China. Pediatr Infect Dis J. 2022;41(1):6–11. 34508026 10.1097/INF.0000000000003338 PMC8658967

[62] Nair D, Thekkur P, Thiagesan R, Vyas A, Paul S, Mishra BK, et al. Implementing timeliness metrics for household contact tracing and TB preventive treatment through TB champions in the public sector, India: an explanatory mixed-methods study. BMJ Open. 2025;15(11):e097935. 41248341 10.1136/bmjopen-2024-097935 PMC12588006

[63] Nair D, Thekkur P, Mbithi I, Khogali M, Zachariah R, Dar Berger S, et al. Timeliness metrics for screening and preventing TB in household contacts of pulmonary TB patients in Kenya. IJTLD Open. 2024;1(1):41–9. 38919414 10.5588/ijtldopen.23.0545 PMC11189597

[64] Jamil B, Nair D, Thekkur P, Laeeq N, Adil A, Khogali M, et al. Feasibility, enablers and challenges of using timeliness metrics for household contact tracing and TB preventive therapy in Pakistan. PLoS ONE. 2023;18(12):e0295580. 38079438 10.1371/journal.pone.0295580 PMC10712885

[65] Mahajan P, Soundappan K, Singla N, Mehta K, Nuken A, Thekkur P, et al. Test and Treat Model for Tuberculosis Preventive Treatment among Household Contacts of Pulmonary Tuberculosis Patients in Selected Districts of Maharashtra: A Mixed-Methods Study on Care Cascade, Timeliness, and Early Implementation Challenges. Trop Med Infect Dis. 2023;9(1):7. 38251204 10.3390/tropicalmed9010007 PMC10818418

[66] Chinese Center for Disease Control and Prevention. Technical Guidelines for Tuberculosis Prevention and Control in China. 2022. https://tb.chinacdc.cn/xxjlg/202111/W020211119672904030470.pdf. Accessed 16 Dec 2025.

[67] Giridharan P, Suseela RP, Zangpo T, Joshi RB, Cader M, Isbaniah F, et al. Tuberculosis preventive treatment in eight SEAR countries - Current practices, implementation challenges and operations research priorities. Public Health Pract (Oxf). 2024;8:100518. 39045000 10.1016/j.puhip.2024.100518 PMC11263745

[68] World Health Organization. Global tuberculosis report 2024. 2024. https://www.who.int/teams/global-programme-on-tuberculosis-and-lung-health/tb-reports/global-tuberculosis-report-2024. Accessed 16 Dec 2025.

[69] Jaswal MR, Martinez L, Brooks M, Farooq S, Safdar N, Shah JA, et al. Tuberculosis-Preventive Treatment for Household Contacts at Health Facility and Community Settings in Pakistan. Clin Infect Dis. 2025;80(6):1290–2. 40036384 10.1093/cid/ciaf088

[70] Nahid P, Mase SR, Migliori GB, Sotgiu G, Bothamley GH, Brozek JL, et al. Treatment of Drug-Resistant Tuberculosis. An Official ATS/CDC/ERS/IDSA Clinical Practice Guideline. Am J Respir Crit Care Med. 2019;200(10):e93–142. 31729908 10.1164/rccm.201909-1874ST PMC6857485

[71] Hasan T, Thu Anh N, Binh Hoa N, Viet Nhung N, Yapa HM, Graham SM, et al. Evaluating the cost-effectiveness of levofloxacin therapy for household contacts of multidrug-resistant tuberculosis in Vietnam. Lancet Reg Health West Pac. 2025;61:101666. 40822287 10.1016/j.lanwpc.2025.101666 PMC12355538

[72] Ryckman T, Weiser J, Gombe M, Turner K, Soni P, Tarlton D, et al. Impact and cost-effectiveness of short-course tuberculosis preventive treatment for household contacts and people with HIV in 29 high-incidence countries: a modelling analysis. Lancet Glob Health. 2023;11(8):e1205–16. 37474228 10.1016/S2214-109X(23)00251-6 PMC10369017

[73] Carratalà-Castro L, Munguambe S, Saavedra-Cervera B, de Haas P, Kay A, Marcy O, et al. Performance of stool-based molecular tests and processing methods for paediatric tuberculosis diagnosis: a systematic review and meta-analysis. Lancet Microbe. 2025;6(6):100963. 39547244 10.1016/j.lanmic.2024.100963 PMC12062341

[74] Seid G, Alemu A, Tsedalu T, Dagne B. Value of urine-based lipoarabinomannan (LAM) antigen tests for diagnosing tuberculosis in children: systematic review and meta-analysis. IJID Reg. 2022;4:97–104. 35880002 10.1016/j.ijregi.2022.06.004 PMC9307507

