# Supplementary material for: Screening and tuberculosis preventive treatment among household contacts of drug-resistant tuberculosis patients: a scoping review
Source: BMC Infect Dis. 2026 Jul 24;26:1438. doi: 10.1186/s12879-026-14056-1 (PMC13450480; doi:10.1186/s12879-026-14056-1)
Supplement: Supplementary file 1 [file 12879_2026_14056_MOESM1_ESM.docx]

**Supplementary Appendix S1. Full Search Strategy for English-language Databases**

The following search strategy for PubMed is presented as a representative example. The same search terms were applied across all English-language databases (PubMed, Embase, Web of Science, Cochrane Library, CINAHL), with adaptations made only to conform to the syntax and interface requirements of each database.

#1 (Contact Tracing[MeSH]) OR (Tracing, Contact[Title/Abstract]) OR (Communicable Disease Contact Tracing[Title/Abstract]) OR (Contact Investigation[Title/Abstract]) OR (Investigation, Contact[Title/Abstract]) OR (Contact Screening[Title/Abstract]) OR (Screening, Contact[Title/Abstract]) OR (Infectious Disease Contact Tracing[Title/Abstract]) OR (Close Contact[Title/Abstract]) OR (family contact[Title/Abstract]) OR (household contact[Title/Abstract]) OR (Household close contacts[Title/Abstract]) OR (Close family contacts[Title/Abstract]) OR (HHC[Title/Abstract]) OR (household member[Title/Abstract]) OR (family[Title/Abstract])

#2 (Extensively Drug-Resistant Tuberculosis[MeSH]) OR (Extensively Drug-Resistant Tuberculosis[Title/Abstract]) OR (Extensively Drug Resistant Tuberculosis[Title/Abstract]) OR (XDR-TB[Title/Abstract]) OR (Extremely Drug-Resistant Tuberculosis[Title/Abstract]) OR (Drug-Resistant Tuberculoses, Extremely[Title/Abstract]) OR (Drug-Resistant Tuberculosis, Extremely[Title/Abstract]) OR (Extremely Drug-Resistant Tuberculoses[Title/Abstract]) OR (Extremely Drug Resistant Tuberculosis[Title/Abstract]) OR (Tuberculoses, Extremely Drug-Resistant[Title/Abstract]) OR (Tuberculosis, Extensively Drug-Resistant[Title/Abstract]) OR (Drug-Resistant Tuberculoses, Extensively[Title/Abstract]) OR (Drug-Resistant Tuberculosis, Extensively[Title/Abstract]) OR (Extensively Drug-Resistant Tuberculoses[Title/Abstract]) OR (Tuberculoses, Extensively Drug-Resistant[Title/Abstract]) OR (Tuberculosis, Extensively Drug Resistant[Title/Abstract]) OR (Tuberculosis, Extremely Drug-Resistant[Title/Abstract]) OR (Tuberculosis, Extremely Drug Resistant[Title/Abstract])

#3 (Tuberculosis, Multidrug-Resistant[MeSH]) OR (Tuberculosis, Multidrug-Resistant[Title/Abstract]) OR (Multidrug-Resistant Tuberculosis[Title/Abstract]) OR (Tuberculosis, Multidrug Resistant[Title/Abstract]) OR (Tuberculosis, MDR[Title/Abstract]) OR (MDR Tuberculosis[Title/Abstract]) OR (Tuberculosis, Multi-Drug Resistant[Title/Abstract]) OR (Multi-Drug Resistant Tuberculosis[Title/Abstract]) OR (Tuberculosis, Multi Drug Resistant[Title/Abstract]) OR (Tuberculosis, Drug-Resistant[Title/Abstract]) OR (Drug-Resistant Tuberculosis[Title/Abstract]) OR (Tuberculosis, Drug Resistant[Title/Abstract]) OR (MDR-TB[Title/Abstract])

#4 (rifampicin-resistant tuberculosis[Title/Abstract]) OR (RR-TB[Title/Abstract]) OR (DR-TB[Title/Abstract])

#5 (Chemoprevention[MeSH]) OR (Chemoprophylaxis[Title/Abstract]) OR (Antibiotic Prophylaxis[Title/Abstract]) OR (Drug Therapy[Title/Abstract])

#6 (Latent Tuberculosis[MeSH]) OR (Latent Tuberculoses[Title/Abstract]) OR (Tuberculoses, Latent[Title/Abstract]) OR (Tuberculosis, Latent[Title/Abstract]) OR (Latent Tuberculosis Infection[Title/Abstract]) OR (Infection, Latent Tuberculosis[Title/Abstract]) OR (Infections, Latent Tuberculosis[Title/Abstract]) OR (Latent Tuberculosis Infections[Title/Abstract]) OR (Tuberculosis Infection, Latent[Title/Abstract]) OR (Tuberculosis Infections, Latent[Title/Abstract]) OR (Latent TB Infection[Title/Abstract]) OR (latent tuberculosis treatment[Title/Abstract])

#7 (Mass Screening[MeSH]) OR (Mass Screenings[Title/Abstract]) OR (Screening, Mass[Title/Abstract]) OR (Screenings, Mass[Title/Abstract]) OR (Screening[Title/Abstract]) OR (Screenings[Title/Abstract])

#8 (preventive therapy[Title/Abstract]) OR (preventive measures[Title/Abstract]) OR (preventive treatment[Title/Abstract]) OR (prevention[Title/Abstract]) OR (IGRA[Title/Abstract]) OR (interferon-gamma release assay[Title/Abstract]) OR (TST[Title/Abstract]) OR (tuberculin skin test[Title/Abstract]) OR (active case finding[Title/Abstract]) OR (GeneXpert[Title/Abstract]) OR (fluoroquinolone prophylaxis[Title/Abstract]) OR (levofloxacin[Title/Abstract]) OR (delamanid[Title/Abstract])

#9 #1 AND (#2 OR #3 OR #4) AND (#5 OR #6 OR #7 OR #8)

**Supplementary Appendix S2. Full Search Strategy for Chinese-language Databases**

The following search strategy for CNKI is presented as a representative example. The same search terms were applied across all Chinese-language databases (SinoMed, CNKI, Wanfang, VIP), with adaptations made only to conform to the syntax and interface requirements of each database.

#1 Title/Abstract/Keywords = contact tracing OR household contact OR close family contact OR close contact OR contact OR cohabitant OR family OR HHC

#2 Subject = MDR-TB OR XDR-TB OR drug-resistant tuberculosis OR drug-resistant TB OR drug-resistant pulmonary TB OR rifampicin-resistant TB OR MDR-TB OR XDR-TB OR RR-TB OR DR-TB

#3 Title/Abstract/Keywords = chemoprevention OR latent tuberculosis OR drug prophylaxis OR screening OR preventive therapy OR latent TB infection OR fluoroquinolone OR levofloxacin OR delamanid OR IGRA OR TST OR GeneXpert

#4 #1 AND #2 AND #3

**Supplementary Table S1. Characteristics of all studies included**

| **First Author** | **Year** | **Study Type** | **Country** | **Age** | **Gender** | **Index Cases** | **No. of HHCs** | | | **Screening Strategy** | | | **TPT Regimen** | | |
| --- | --- | --- | --- | --- | --- | --- | --- | --- | --- | --- | --- | --- | --- | --- | --- |
|  |  |  |  |  |  |  | **Total** | **Children** | **HIV+** | **Method** | **Coverage** | **Outcomes** | **Drug(s)** | **Dose** | **Duration** |
| Yang, H. [39] | 2023 | Case Series | China | NR | NR | 650 | 1352 | NR | NR | Symptom screening; CXR; AFB smear; Culture; TST; IGRA | 81.4% | Active TB: 1.4% | NR | NR | NR |
| Zhou, Y. [34] | 2023 | Cross-sectional | China | 3-25y: 22.0%; 26-64y: 72.0%;  65-71y: 6.0% | F: 56.7%; M: 43.3% | 166 | 282 | NR | NR | Symptom screening; CXR; AFB smear; Culture; TST; DST; Genotyping | NR | LTBI: 37.6%; Active TB: 1.4% | NR | NR | NR |
| Krishnan, S. [38] | 2023 | Prospective cohort | 8 countries* | ≤5y: 10%;  5-<15y: 20%; 15-<45y:47%;  6-≥45y: 23% | F: 59%; M: 41% | 308 | 1016 | <=5y: 103; 5-<15y: 201 | 65 (6%) | Symptom screening; CXR; AFB smear; Culture; Gastric aspirate; GeneXpert MTB/RIF; IGRA | 83.7% | 1-yr TBI: 21.6%; TBD: 2.3%; Confirmed TBD: 1.1% | INH (n=12); INH/LFX/EMB (n=5); INH/MFX/ETH (n=2);  unknown (n=2) | NR | NR |
| Gupta, A. [28] | 2020 | Cross-sectional | 8 countries* | <5y: 10%;  5-15y: 20%; 15-50y: 53%;  ≥50y: 17% | F: 59%; M: 41% | 284 | 1007 | <15y: 303 (30%); <5y: 102 (10%) | 65 (6%) | Symptom screening; CXR; AFB smear; Culture; Gastric aspirate; GeneXpert MTB/RIF; TST; IGRA; HIV testing | 79% | LTBI: 72%; Active TB: 12% | INH or INH/EMB/FQ | NR | NR |
| Iqbal, Z. [41] | 2023 | Cross-sectional | Pakistan | <15y: 7.0%; 15-44y:59.5%;  45-64y: 27.1%;  ≥65y: 6.4% | F: 60.9%; M: 39.1% | 145 | 1310 | <15y: 92 (7.0%) | NR | Symptom screening; GeneXpert MTB/RIF | NR | Active TB: 5.95% | NR | NR | NR |
| Kendall, M.A. [26] | 2025 | Phase 3 multicenter RCT | 13 countries** | All ages | NR | NR | 3834 | NR | NR | Symptom screening; CXR; AFB smear; Culture; Gastric aspirate; GeneXpert MTB/RIF; TST; IGRA; DST; HIV testing; ECG | NR | Results pending | Delamanid vs. INH + Pyridoxine | Delamanid: weight-based (30-200 mg/d); INH: weight-based (50-300 mg/d); Pyridoxine: per local guidelines | 26 weeks |
| Fox, G.J. [48] | 2024 | Double-blind RCT | Vietnam | All ages | F: 64%; M: 36% | NR | 3948 | <15y: 60 (2.9%) | 8 (0.4%) | Symptom screening; CXR; AFB smear; Culture; GeneXpert MTB/RIF; TST | NR | LTBI: 99.8%; Active TB: 0.6% (LFX) vs. 1.1% (placebo) | LFX | Adults: 10-15 mg/kg/d;  Children: 15-20 mg/kg/d; max 750 mg/d | 6 months |
| Schaaf, H.S. [29] | 1999 | Prospective cohort | South Africa | ≤5 years | F: 54%; M: 46% | 75 | 128 | 128 (100%) | 1 | CXR; Culture; Gastric aspirate; TST; HIV testing; ESR; Clinical assessment | NR | LTBI: 51.6%; Active TB: 11.7% | INH + PZA + ETH +EMB (per adult DST) | INH 15-20 mg/kg/d; PZA 25-35 mg/kg/d; ETH 10-15 mg/kg/d; EMB 15-20 mg/kg/d | Prophylaxis 6 months; Treatment 9-12 months |
| Kim, S. [49] | 2023 | Cross-sectional | 8 countries* | ≥15 years | F: 63%; M: 37% | 279 | 712 | 0 | 59 (8%) | Symptom screening; CXR; AFB smear; Culture; GeneXpert MTB/RIF; IGRA | NR | LTBI: 68.8%; Active TB: 6.5% | TPT not provided | - | - |
| Apolisi, I. [30] | 2023 | Prospective cohort | South Africa | 0-18 years | F: 51%; M: 49% | 48 | 112 | 112 (100%) | 6 (5%) | Symptom screening; CXR; AFB smear; Gastric aspirate; Stool Xpert Ultra; HIV testing; Physical exam | 77% | Active TB: 9.8% | LFX (83.2%); INH (9.5%); Delamanid (7.4%) | Standard weight-based dosing | 6 months, once daily |
| Qadeer, E. [42] | 2017 | Cross-sectional | Pakistan | Median: 24y (IQR 14-38) | F: 46%; M: 54% | 209 | 1467 | <5y: 95;  5-15y:282;  Total: 377 (25.7%) | NR | Symptom screening; CXR; AFB smear; GeneXpert MTB/RIF | NR | Active TB: 3.8% | TPT not provided | - | - |
| Malik, A.A. [31] | 2021 | Prospective cohort | Pakistan | <15y: 36%;  ≥15y: 64% | F: 46%; M: 54% | 100 | 800 | <15y: 128 (74%); <5y: 35% | NR | Symptom screening; CXR; AFB smear; TST; HIV testing | 93.1% | Active TB: 1.2% | LFX/EMB; LFX/ETH; MFX/EMB; MFX/ETH | LFX (<5y: 15-20 mg/kg; >5y: 7.5-10 mg/kg; max 1000 mg/d); MFX (7.5-10 mg/kg; max 400 mg/d); EMB (15-25 mg/kg; max 2000 mg/d); ETH (15-20 mg/kg; max 750 mg/d) | 6 months |
| Huerga, H. [32] | 2019 | Prospective cohort | Armenia | Children <15y (<2y: 16%;  2-4y: 24%;  >5y: 60%) | F: 53.3%; M: 46.7% | 79 | 150 | <2y: 24;  2-4y: 36;  >5y: 90 | NR | CXR; TST; IGRA; Stool Xpert Ultra; Clinical assessment | 75.8% | LTBI: 58.7%; Active TB: 2.0% | TPT not provided | - | - |
| Rekart, M.L. [47] | 2023 | Retrospective cohort | Tajikistan | All ages | F: 57.6%; M: 42.4% | 830 | 6654 | <5y: 1363 | 2 | Symptom screening; CXR; Culture; GeneXpert MTB/RIF; TST; IGRA; DST | NR | Active TB: 0.7% | TPT not provided | - | - |
| Schaaf, H.S. [35] | 2002 | Prospective cohort (30-month follow-up) | South Africa | ≤5 years | F: 54%; M: 46% | 73 | 125 | 125 (100%) | 1 | CXR; Culture; Gastric aspirate; TST; HIV testing; ESR; Physical exam; History taking | 92.8% | LTBI: 53%; Active TB: 11% | INH + PZA + ETH + EMB + OFX | INH 15-20 mg/kg/d; PZA 25-35 mg/kg/d; ETH 10-15 mg/kg/d; EMB 15-20 mg/kg/d; OFX 15 mg/kg/d | 6 months |
| Amanullah, F. [36] | 2014 | Cross-sectional | Pakistan | 0-5 years;  5-14 years | F: 53.4%; M: 46.6% | 227 | 133 | <15y: 133 (100%) | NR | CXR; Chest CT; AFB smear; Culture; Gastric aspirate; TST; DST; HIV testing; ESR; CBC; Physical exam; History taking | NR | LTBI: 43.5%; Active TB: 7.5% | TPT not provided | - | - |
| Shi, Z. [37] | 2025 | Cross-sectional | China | 3-78 years | F: 57.2%; M: 42.8% | 197 | 264 | NR | NR | Symptom screening; CXR; Chest CT; TST; IGRA; ESAT6-CFP10 skin test | NR | LTBI: 32.2%; Active TB: 9.8% | TPT not provided | - | - |
| Swindells, S. [50] | 2018 | Cross-sectional | 8 countries* | <5y: 13%;  ≥5y: 87% | F: 59%; M: 41% | 308 | 1017 | <5y: 103; <18y: 354 | 39 (4%) | Symptom screening; CXR; AFB smear; Culture; GeneXpert MTB/RIF; TST; IGRA | 99.9% | LTBI (TST+): 56%; LTBI (IGRA+): 65%; Active TB: 12% | NR | NR | NR |
| Shadrach, B.J. [43] | 2021 | Cross-sectional | India | <18y: 56.8%;  >18y: 43.2% | F: 40.2%; M: 59.8% | 87 | 271 | <18y: 154 (56.8%) | 2 | Symptom screening; CXR; AFB smear; GeneXpert MTB/RIF; TST; DST; HIV testing | 95.1% | LTBI: 45.8%; Active TB: 35.8% | Isoniazid monotherapy | NR | 6 months |
| Malik, A.A. [40] | 2020 | Prospective cohort | Pakistan | Median: 19y (IQR 10-32) | F: 42.9%; M: 53.1% | 100 | 800 | <5y: 94 | NR | Symptom screening; CXR; AFB smear; GeneXpert MTB/RIF; TST; CBC; Physical exam | 92.1% | Active TB: 1.4% | LFX or MFX + EMB or ETH | LFX (<=5y: 15-20 mg/kg; >5y: 7.5-10 mg/kg; max 1000 mg/d); MFX (7.5-10 mg/kg; max 400 mg/d); EMB (15-25 mg/kg; max 2000 mg/d); ETH (15-20 mg/kg; max 750 mg/d) | 6 months |
| Seddon, J.A. [27] | 2018 | Phase III cluster RCT | South Africa | <5 years | All genders | 778 | 1556 | 1556 (100%) | NR | Symptom screening; CXR; AFB smear; Culture; GeneXpert MTB/RIF; IGRA; HIV testing | NR | NR | LFX vs. placebo | 15-20 mg/kg/d, once daily | 24 weeks (6 months) |
| Tsogt, B. [45] | 2025 | Prospective implementation | Mongolia | All ages | F: 49.0%; M: 51.0% | 99 | 349 | <15y: 133 | NR | Symptom screening; CXR; GeneXpert MTB/RIF; TST | 99.4% | LTBI: 40%; Active TB: 4.9% | LFX | NR | 6 months |
| Kyaw, N.T.T. [46] | 2019 | Cross-sectional | Myanmar | Median: 31y (IQR 16-46) | F: 58.4%; M: 41.6% | 210 | 620 | <5y: 40;  5-14y: 98; Total: 138 | 6 (1.0%) | Symptom screening; CXR; AFB smear; GeneXpert MTB/RIF | Symptom: 100%; CXR: 81%; Sputum: 70% | Active TB: 3.9% | NR | NR | NR |
| Javaid, A. [44] | 2016 | Cross-sectional | Pakistan | <15y: 10.2%; 15-44y: 75.2%; 45-64y: 12.3%;  >65y: 2.3% | F: 58.4%; M: 41.6% | 154 | 610 | <15y: 62 (10.2%) | NR | Symptom screening; CXR; AFB smear; GeneXpert MTB/RIF | 77% | MDR-TB: 17.4%; DS-TB: 4.2% | NR | NR | NR |
| Duong, T. [33] | 2025 | Double-blind multisite RCT | South Africa | <1y: 18%;  1-3y: 34%;  3-5y: 39%;  5-10y: 4%;  10-18y: 5% | F: 51%; M: 49% | 497 | 922 | 911 (98.8%) | 19 (2%) | Symptom screening; CXR; AFB smear; Culture; GeneXpert MTB/RIF; IGRA; HIV testing; Physical exam | NR | LTBI: 27%; Active TB: 1.1% (LFX) vs. 2.6% (placebo) | LFX vs. placebo | 15-20 mg/kg/d; max 750 mg/d, once daily | 24 weeks |
| Malik, A.A. [51] | 2021 | Prospective cohort | Pakistan | <5y: 35%;  5-9y: 26%;  10-19y: 28%;  >19y: 11% | F: 47%; M: 53% | 100 | 215 | <5y: 61 (35%) | NR | CXR; GeneXpert MTB/RIF; TST; Physical exam; History taking | 80% | Active TB: 0% | LFX or MFX + EMB or ETH | LFX (<=5y: 15-20 mg/kg; >5y: 7.5-10 mg/kg; max 1000 mg/d); MFX (7.5-10 mg/kg; max 400 mg/d); EMB (15-25 mg/kg; max 2000 mg/d); ETH (15-20 mg/kg; max 750 mg/d) | 6 months |

** 8 countries: Botswana, Brazil, Haiti, India, Kenya, Peru, South Africa, Thailand; ** 13 countries: Botswana, Brazil, Philippines, Tanzania, Zimbabwe, Haiti, India, Kenya, Uganda, Vietnam, Peru, Thailand, South Africa. Abbreviations: NR: Not reported; HHCs, household contacts; LTBI, latent tuberculosis infection; TB, tuberculosis; TBI, tuberculosis infection; TBD, tuberculosis disease; MDR-TB, multidrug-resistant TB; DS-TB, drug-susceptible TB; INH, isoniazid; PZA, pyrazinamide; EMB, ethambutol; ETH, ethionamide; LFX, levofloxacin; MFX, moxifloxacin; OFX, ofloxacin; FQ, fluoroquinolone; RCT, randomised controlled trial; F, female; M, male; y, years; IQR, interquartile range; CXR, chest radiography; AFB, acid-fast bacilli; TST, tuberculin skin test; IGRA, interferon-gamma release assay; DST, drug-susceptibility testing; ECG, electrocardiogram; ESR, erythrocyte sedimentation rate; CBC, complete blood count.*

**Supplementary Table S2. JBI Critical Appraisal Results for Cohort Studies (n = 10)**

| **Appraisal item** | **Krishnan, S. [38]** | **Schaaf, H.S. [29]** | **Apolisi, I. [30]** | **Malik, A.A. [31]** | **Huerga, H. [32]** | **Rekart, M.L. [47]** | **Schaaf, H.S. [35]** | **Malik, A.A. [40]** | **Tsogt, B. [45]** | **Malik, A.A. [51]** |
| --- | --- | --- | --- | --- | --- | --- | --- | --- | --- | --- |
| 1. Were the two groups similar and recruited from the same population? | Yes | Unclear | N/A | N/A | N/A | N/A | Unclear | Unclear | N/A | Unclear |
| 2. Were the exposures measured similarly to assign people to both exposed and unexposed groups? | Yes | Yes | N/A | N/A | N/A | N/A | Yes | Yes | N/A | Yes |
| 3. Was the exposure measured in a valid and reliable way? | Yes | Yes | Yes | Yes | Yes | Yes | Yes | Yes | Yes | Yes |
| 4. Were confounding factors identified? | Yes | Yes | Yes | Yes | Yes | Yes | Yes | Yes | Yes | Yes |
| 5. Were strategies to deal with confounding factors stated? | Yes | Yes | Yes | Unclear | Yes | Unclear | Yes | Unclear | Unclear | Yes |
| 6. Were the groups/participants free of the outcome at the start of the study (or at the moment of exposure)? | Yes | Unclear | Yes | Yes | Yes | Yes | Unclear | Yes | Yes | Yes |
| 7. Were the outcomes measured in a valid and reliable way? | Yes | Yes | Yes | Yes | Yes | Yes | Yes | Yes | Yes | Yes |
| 8. Was the follow up time reported and sufficient to be long enough for outcomes to occur? | Yes | Yes | Yes | Yes | Yes | Yes | Yes | Yes | Yes | Yes |
| 9. Was follow up complete, and if not, were the reasons to loss to follow up described and explored? | Yes | Yes | Yes | Unclear | Yes | No | No | Yes | Yes | Unclear |
| 10. Were strategies to address incomplete follow up utilized? | Yes | N/A | Unclear | Yes | Yes | Yes | Yes | Unclear | Yes | Yes |
| 11. Was appropriate statistical analysis used? | Yes | Unclear | Yes | Yes | Yes | Yes | Yes | Yes | Yes | Yes |

*N/A, not applicable.*

**Supplementary Table S3. JBI Critical Appraisal Results for Cross-Sectional Studies (n = 11)**

| **Appraisal item** | **Zhou, Y. [34]** | **Gupta, A. [28]** | **Iqbal, Z. [41]** | **Kim, S. [49]** | **Qadeer, E. [42]** | **Amanullah, F. [36]** | **Shi, Z. [37]** | **Swindells, S. [50]** | **Shadrach, B.J. [43]** | **Kyaw, N.T.T. [46]** | **Javaid, A. [44]** |
| --- | --- | --- | --- | --- | --- | --- | --- | --- | --- | --- | --- |
| 1. Were the criteria for inclusion in the sample clearly defined? | Yes | Yes | Unclear | Yes | Yes | Yes | Yes | Yes | Yes | Yes | Yes |
| 2. Were the study subjects and the setting described in detail? | Yes | Yes | Yes | Yes | Yes | Yes | Yes | Yes | Yes | Yes | Yes |
| 3. Was the exposure measured in a valid and reliable way? | Yes | Yes | Yes | Yes | Unclear | Yes | Yes | Yes | Yes | Yes | Yes |
| 4. Were objective, standard criteria used for measurement of the condition? | Yes | Yes | Yes | Yes | Yes | Yes | Yes | Yes | Yes | Yes | Yes |
| 5. Were confounding factors identified? | No | Yes | No | Yes | Unclear | Unclear | Yes | Unclear | Unclear | Yes | No |
| 6. Were strategies to deal with confounding factors stated? | No | Yes | No | Yes | Yes | Unclear | Yes | No | Yes | Yes | No |
| 7. Were the outcomes measured in a valid and reliable way? | Yes | Yes | Yes | Yes | Yes | Yes | Yes | Yes | Yes | Yes | Yes |
| 8. Was appropriate statistical analysis used? | Unclear | Yes | No | Yes | Yes | Yes | Yes | Yes | Yes | Yes | No |

*N/A, not applicable.*

**Supplementary Table S4. JBI Critical Appraisal Results for Randomized Controlled Trials (n = 2)**

| **Appraisal item** | **Fox, G.J. [48]** | **Duong, T. [33]** |
| --- | --- | --- |
| 1. Was true randomization used for assignment of participants to treatment groups? | Yes | Yes |
| 2. Was allocation to treatment groups concealed? | Yes | Yes |
| 3. Were treatment groups similar at the baseline? | Yes | Yes |
| 4. Were participants blind to treatment assignment? | Yes | Yes |
| 5. Were those delivering treatment blind to treatment assignment? | Yes | Yes |
| 6. Were outcomes assessors blind to treatment assignment? | Yes | Yes |
| 7. Were treatment groups treated identically other than the intervention of interest? | Yes | Yes |
| 8. Was follow up complete and if not, were differences between groups in terms of their follow up adequately described and analyzed? | Yes | Yes |
| 9. Were participants analyzed in the groups to which they were randomized? | Yes | Yes |
| 10. Were outcomes measured in the same way for treatment groups? | Yes | Yes |
| 11. Were outcomes measured in a reliable way? | Yes | Yes |
| 12. Was appropriate statistical analysis used? | Yes | Yes |
| 13. Was the trial design appropriate, and any deviations from the standard RCT design (individual randomization, parallel groups) accounted for in the conduct and analysis of the trial? | Yes | Yes |

**Supplementary Table S5. JBI Critical Appraisal Results for Case Series (n = 1)**

| **Appraisal item** | **Yang, H. [39]** |
| --- | --- |
| 1. Were there clear criteria for inclusion in the case series? | Yes |
| 2. Was the condition measured in a standard, reliable way for all participants included in the case series? | Yes |
| 3. Were valid methods used for identification of the condition for all participants included in the case series? | Yes |
| 4. Did the case series have consecutive inclusion of participants? | Yes |
| 5. Did the case series have complete inclusion of participants? | Unclear |
| 6. Was there clear reporting of the demographics of the participants in the study? | Yes |
| 7. Was there clear reporting of clinical information of the participants? | Yes |
| 8. Were the outcomes or follow up results of cases clearly reported? | Yes |
| 9. Was there clear reporting of the presenting site(s)/clinic(s) demographic information? | Yes |
| 10. Was statistical analysis appropriate? | Yes |

**Supplementary Table S6. Implementation barriers and facilitating factors at different levels**

| **Level** | **Implementation barriers** | **Facilitating factors** |
| --- | --- | --- |
| **Patient / Family Level** | Refusal of screening or treatment by contacts.  Pregnant or breastfeeding women discontinuing medication independently.  Poor adherence attributed to adverse drug reactions, prolonged treatment duration, daily pill burden, and absence of perceived symptoms.  Difficulty obtaining sputum or blood samples from young children; guardians more likely to refuse blood testing.  Pediatric adherence compromised by large tablet size, unpleasant taste, and need for forced administration.  Asymptomatic children or adolescents show low healthcare-seeking behavior.  Caregiver competing responsibilities leading to treatment discontinuation.  Sociocultural factors including reliance on herbal/traditional medicine (independent predictor of early discontinuation). | Decentralized service model with trained community volunteers or community health workers conducting home visits to assist children.  Transportation subsidies and provision of free medications to reduce financial burden.  Family education and psychological support. |
| **Health Service Provider Level** | Insufficient knowledge or confidence among healthcare providers.  Limited diagnostic capacity for childhood tuberculosis; lack of pediatric-specific diagnostic protocols and preventive treatment guidelines.  Limited use of gastric aspirate or induced sputum for microbiological evaluation in children even when indicated.  DST results from index cases not consistently applied to optimize pediatric preventive regimens.  Unstable supply of medicines and reagents. | Enhanced training and counseling for providers.  Regular psychological follow-up and adverse event monitoring.  Periodic home visits by community workers or dedicated nurses.  Use of online coordination platforms and electronic medication monitoring devices. |
| **Health System Level** | Resource constraints: limited workforce, equipment shortages, and high X-ray screening costs.  Low screening uptake and TPT coverage.  Low sensitivity of symptom-based screening, leading to risk of missed diagnoses.  Inability to trace all contacts.  Transportation and logistical barriers (long distances, high costs, long waiting times). | Active contact tracing mechanisms.  National Tuberculosis Program (NTP) covering the entire DR-TB management process.  Research collaborations with local projects (e.g., PHOENIx trial).  Establishment of household contact database and multicenter collaborative network with unified data management. |
| **Community / Policy Level** | Stigmatization of tuberculosis among patients, communities, and healthcare facilities.  Poor household conditions: overcrowding, inadequate ventilation, and poverty.  Some national guidelines do not include TPT for DR-TB contacts. | Community education and outreach programs.  Engagement of community health workers and volunteers.  National-level planning support and institutional safeguards.  Oversight of program platforms to ensure scientifically sound design. |

*Abbreviations: TPT, tuberculosis preventive treatment; DR-TB, drug-resistant tuberculosis; DST, drug-susceptibility testing; NTP, National Tuberculosis Program.*
